# Comparison of Metabolome and Functional Properties of Three Korean Cucumber Cultivars

**DOI:** 10.3389/fpls.2022.882120

**Published:** 2022-04-15

**Authors:** Hyo Eun Jo, Su Young Son, Choong Hwan Lee

**Affiliations:** ^1^Department of Bioscience and Biotechnology, Konkuk University, Seoul, South Korea; ^2^Research Institute for Bioactive-Metabolome Network, Konkuk University, Seoul, South Korea

**Keywords:** cucumber, fruit quality, metabolomics, antioxidant activity, gene expression, carotenoid and chlorophyll metabolism

## Abstract

Cucumber (*Cucumis sativus* L.) is consumed worldwide and various cultivars have been developed to enhance fruit quality. However, few studies have comprehensively evaluated the quality of various cultivars. We carried out a metabolomics approach to study the three different cucumber cultivars (Chuichung, White Dadagi, and Mini) and their parts (peel and flesh) coupled with antioxidant activities. The amino acids, sugars, flavonoids, carotenoids, and chlorophylls were upregulated in Mini flesh; however, in the case of peel, they were highly expressed in Chuichung. The highest antioxidant activity was observed in the peel of Chuichung and flesh of Mini. Through correlation analysis between metabolites and antioxidant activity, apigenin and quercetin derivatives, chlorophyll a, chlorophyll b, lutein, α-carotene, and β-carotene were found to be significantly positively correlated with antioxidant activity. To understand the metabolism of these compounds, we performed a comprehensive pathway analysis using a metabolomics approach and analysis of associated gene expression. In secondary metabolism, the expression levels of carotenoid-related genes (15-*cis*-phytoene synthase and ζ-carotene desaturase) and chlorophyll-related genes (protochlorophyllide reductase and glutamyl-tRNA reductase) were consistent with the metabolome analysis data. Collectively, carotenoid and chlorophyll metabolism were upregulated in Chuichung peel and Mini flesh, which had the highest antioxidant activity in each part. These bioactive compounds can be used as biomarkers of commercial cucumber fruit quality. Accordingly, this study offers integrative insights into the quality of different cucumber cultivars and explores valuable metabolites and genes that are helpful in improving quality with functional properties.

## Introduction

Cucumber (*Cucumis sativus*) is a widely cultivated and consumed vegetable around the world. Although over 90% of fresh cucumber fruit is water, it is an important commercial resource because it also contains various bioactive compounds associated with multiple biological properties, including antioxidant, anti-wrinkle, anti-aging, and antimicrobial activities (Kamkaen et al., 2007; Sotiroudis et al., 2010; Nema et al., 2011). In particular, antioxidant activity is nutritionally important for human health. Previous studies have shown that secondary metabolites, including flavonoids, carotenoids, and chlorophylls, affect antioxidant activity (Heim et al., 2002; Pérez-gálvez et al., 2020). Chlorophylls and carotenoids are pigment molecules that determine the color of cucumber fruit (Wang et al., 2020). Chlorophylls are tetrapyrrole compounds that are green in color, and carotenoids are tetraterpenoids related to yellow, orange-red, and red colors. Cucumber has a unique flavor due to several volatile compounds, known as “cucumber aldehydes,” including (*E,Z*)-2,6-nonadienal and (*E*)-2-nonenal (Buescher and Buescher, 2001). This greatly affects consumer preference for cucumber fruit, along with its morphological traits, tastes, and compounds in relation to the activities mentioned above. These are crucial factors in determining the quality of cucumber fruit.

Recently, different cultivars of cucumbers have been developed and introduced into the market to meet the diverse consumer preferences. Studies on the metabolism, transcription, and activity of cucumbers have been conducted; however, integrated research remains limited. Few studies have compared commercial cucumbers in detail by dividing them into peel and flesh. Here, an integrated study into cucumber fruit quality was designed using a metabolomics approach.

South Korea is the 16th largest producer of cucumber in the world, with three major cultivar groups being grown: the Baekdadagi-type, Nakhap-type, and Gasi-type cultivars (Park et al., 2021). In addition, nowadays, the demand for mini vegetables has been increasing due to the increase in single-person households. In this study, we aimed to obtain the insights into the fruit quality of cucumber cultivars by constructing metabolome and transcriptome analysis for three commercial Korean cucumbers (Chuichung, White Dadagi, and Mini). These three cultivars are easily available in the Korean local market. The Chuichung and Mini used in this study belong to the Nakhap-type, and White Dadagi belongs to the Baekdadagi-type. Chuichung (Nakhap-type) and White Dadagi (Baekdadagi-type) have been traditionally eaten in Korea for a long time and they are largely distinguished from each other in morphology. The Mini is a new cultivar recently developed and introduced in Korea. Although the Mini belongs to Nakhap-type, but the size is differ to compare traditional cultivars (Chuichung and White Dadagi). The difference of genome between Nakhap-type and Baekdadagi-type has been reported in previous studies (Song et al., 2021). However, few studies have comprehensively evaluated the quality of these cultivars in terms of metabolomics and bioactivity. Thus, we conducted research based on metabolomics approach to interpret difference in morphology and antioxidant activities. This research was intended to analyze the difference among the three cucumber cultivars that were easily obtained and mainly consumed in Korea. Moreover, we tried to provide morphological characteristics and detailed information, such as metabolites, that contribute to fruit odor, flavor, and antioxidant activity of three different cucumber cultivars.

We performed non-targeted metabolomics analysis using gas chromatography time-of-flight mass spectrometry (GC-TOF-MS), ultrahigh-performance liquid chromatography–linear trap quadrupole-orbitrap–tandem mass spectrometry (UHPLC-LTQ-Orbitrap-MS/MS), and headspace-solid phase microextraction gas chromatography time-of-flight mass spectrometry (HS-SPME-GC-TOF-MS) platforms. In addition, different metabolite contents and differentially expressed genes (DEGs) in different cucumber samples were described in a metabolic pathway map. Applying a combination of integrated metabolomic data and its related gene expression data could help us understand the metabolism and molecular mechanisms of gene regulation through the construction of a single pathway.

This study presents a comprehensive picture of the differences in the chemical composition of the primary, secondary, and volatile compounds and the antioxidant activities of three different commercial cucumber fruit. The aim is to provide insights into the metabolism of different cucumber cultivars to facilitate future improvements in cucumber fruit quality.

## Materials and Methods

### Chemicals and Reagents

HPLC-grade solvents were purchased from Fisher Scientific (Waltham, MA, United States). All standard compounds and analytical-grade reagents used in this study were obtained from Sigma-Aldrich (St. Louis, MO, United States) and Junsei Chemical (Tokyo, Japan).

### Sample Preparation

**Figure 1:**
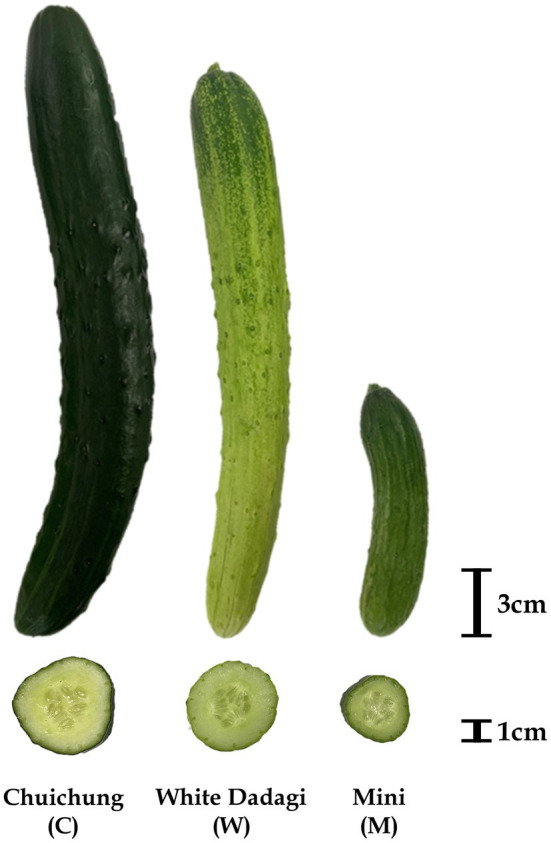
Photographs of representative ripe fruit of the three cucumber cultivars evaluated in this study: Chuichung, White Dadagi, and Mini.

The fruit of three commercial cucumbers (Chuichung, White Dadagi, and Mini) at the ripe stage were purchased from a local market (Seoul, Korea; Figure 1). Each cucumber fruit was washed with distilled water and divided into three segments based on length. In this study, intermediate segments were used for further analyses. Furthermore, the peel and flesh of the cucumber were separated using a hand-held vegetable peeler. Each sample was dried using a freeze dryer (Operon, Gimpo, Korea) for 5 days and then ground into a powder with a mortar and pestle. Powdered cucumber peel and flesh were stored at −80°C until metabolite extraction. Three biological replicates of cucumber peel and flesh were used.

### Sample Extractions for Antioxidant Assays, Total Phenolic, and Flavonoid Contents Analysis, and Metabolic Profiling

Dried powder samples (100 mg) were extracted with 1 ml of 80% aqueous methanol, including internal standard solution (2-chloro-*L*-phenylalanine, 1 mg/ml in water) using an MM 400 mixer mill (Retsch®; Haan, Germany) at a frequency of 30 s^−1^ for 10 min, followed by 5 min of sonication at 4°C (Hettich Zentrifugen Universal 320; Tuttlingen, Germany). The extracted samples were centrifuged at 15,000 rpm for 10 min at 4°C, and the supernatants were filtered using 0.2-μm polytetrafluoroethylene (PTFE) syringe filters (Chromdisc, Daegu, Korea). The filtered supernatants were completely dried using a speed vacuum concentrator (Biotron, Seoul, Korea) and re-dissolved in 80% methanol to obtain a final concentration of 10,000 ppm (10 mg/ml) for the bioactivity assays and instrument analyses.

### Determination of Antioxidant Activity

Antioxidant activities, including 2,2′-azino-bis (3-ethyl benzothiazoline-6-sulfonic acid; ABTS) and ferric reducing antioxidant power (FRAP) radical scavenging assays, were performed using the methods described by Lee et al. (2016) with some modifications.

ABTS solution (7 mM) was dissolved in a 2.45 mM potassium persulfate solution. Then, the mixture was incubated for 20 min at 60°C in the dark and stored overnight at 4°C. The solution was diluted with distilled water until the absorbance reached 0.7 ± 0.02 at 750 nm using a spectrophotometer (SpectraMax®190; Molecular Devices, San Jose, CA, United States). Sample extracts (10 μl) were mixed with 190 μl of diluted ABTS solution in 96-well plates and incubated for 7 min at 37°C in the dark. The absorbance of the reacted samples was recorded at 750 nm using a spectrophotometer (SpectraMax®190; Molecular Devices, San Jose, CA, United States).

The FRAP solution was mixed with 300 mM acetate buffer (pH 3.6), 10 mM TPTZ(2,4,6-tris(2-pyridyl)-s-triazine; in 40 mM HCl solution), and 20 mM FeCl_3_…6H_2_O (in distilled water) at a ratio of 10:1:1. The assays were performed by adding 10 μl of the sample extract to 300 μl of the FRAP solution in 96-well plates, followed by incubation for 6 min at 37°C in the dark. The absorbance was measured at 570 nm using a spectrophotometer (SpectraMax®190; Molecular Devices, San Jose, CA, United States).

Analysis for antioxidant activities, such as ABTS and FRAP assay, were conducted in triplicate, and the results were displayed as Trolox equivalent antioxidant capacity (TEAC). The concentration ranges were 0.0156–1 and 0.0156–2 mM in the ABTS and FRAP assays, respectively.

### Determination of Total Phenolic and Flavonoid Contents

Total phenolic content (TPC) and total flavonoid content (TFC) were determined using a method described in our previous study (Lee et al., 2016) with some modifications.

To determine the TPC, 20 μl of the sample extracts from each part of the cucumber sample were mixed with 100 μl of 0.2 N Folin–Ciocalteu’s phenol reagent in 96-well plates under dark conditions. After incubation for 6 min, 80 μl of 7.5% Na_2_CO_3_ solution (in distilled water) was added to the mixture. Absorbance was measured at 750 nm using a spectrophotometer (SpectraMax®190; Molecular Devices, San Jose, CA, United States). The results are presented as gallic acid equivalent concentrations, with a concentration range of 3.90625–500 ppm.

To measure TFCs, 180 μl of 90% diethylene glycol (in distilled water), 20 μl of 1 N NaOH, and 20 μl of each sample extract were added to 96-well plates and incubated for 60 min at room temperature in the dark. Lastly, the absorbance was measured at 405 nm using a spectrophotometer (SpectraMax®190; Molecular Devices, San Jose, CA, United States). The results are presented as naringin equivalent concentrations, with a concentration range of 1.5625–200 ppm. All assays were performed in triplicate.

### Analysis of Chlorophylls and Carotenoids Contents

The chlorophylls and carotenoids contents were measured according to the procedures described by Xie et al. (2019) and Wang et al. (2020) with some modifications. Dried powder sample (100 mg) was placed in a 15 ml centrifuge tube with 5 ml of solution (9:1 = acetone: 0.1 M NH_4_OH). After vortexing, the mixture was sonicated for 15 min and centrifuged at 3,000 rpm for 20 min. Supernatants were collected in a 50 ml tube. The same process was repeated in triplicate, and the supernatants were collected using hexane. The absorbance of the mixed supernatant was measured at 662, 645, and 470 nm using a spectrophotometer (SpectraMax®190; Molecular Devices, San Jose, CA, United States). The measurements were performed using three biological replicates.

The chlorophylls and carotenoids contents were calculated as follows:


$$C_{a}=11.75A_{662}–2.35A_{645}$$

$$C_{b}=18.61A_{645}–3.96A_{662}$$

$$C_{ca}=\left(1000A_{470}–2.27C_{a}-81.4C_{b}\right)/227$$

where C_a_, C_b_, and C_ca_ indicate the chlorophyll a, chlorophyll b, and total carotenoids content (μg/mL), respectively, and A_662_, A_645_, and A_470_ represent the absorbances at 662, 645, and 470 nm, respectively.

### Liquid Chromatography-Diode Array Detection (LC-DAD) Analysis for Carotenoids

Carotenoids were extracted from 100 mg of dried powder samples by adding 3 ml of ethanol containing 0.1% ascorbic acid. Mixture vortexed and placed in a water bath at 85°C for 5 min. The carotenoids extract was saponated with 120 μl of 80% potassium hydroxide at 85°C for 10 min. After saponification, the samples were placed on ice and 1.5 ml of cold water was mixed with the sample. β-Apo-8′-carotenal (25 μg/ml) was added as the internal standard. Carotenoids were extracted twice using 1.5 ml of hexane by centrifuging at 1,200 rpm to separate the layers. The supernatants were dried and re-dissolved in 50:50 (v/v) dichloromethane/methanol for analysis. Carotenoids were analyzed using a LC-DAD system, consisting of a Shimadzu Nexera X2 LC-30 AD pump, Shimadzu SIL-30 AC Autosampler, and Shimadzu SPD-20A. Chromatographic separation was performed using a YMC carotenoid C30 column (250 mm × 4.6 mm, 5 μm particle size; YMC, Gyeonggi-do, Korea) with an injection volume of 10 μl. The flow rate was 1 ml/min. The binary solvent system consisted of solvent A (methanol/water (92:8, v/v) with 10 mM ammonium acetate) and solvent B (*tert-butyl* methyl ether). The gradient parameters were set as follows: 0–1 min, 20% B; 1–19 min, 20–100% B; 19–20 min, 100% B; 20–21 min, 100–20% B. Absorbance was measured at a wavelength of 450 nm. Carotenoids were identified by comparing their retention times and absorbance spectra with those of standard carotenoid compounds.

### Metabolome Analysis

#### Gas Chromatography Time-of-Flight Mass Spectrometry Analysis for Primary Metabolites

The dried extracts were re-dissolved in 80% MeOH (10,000 ppm) and filtered using a 0.2-μm PTFE filter (Chromdisc, Daegu, Korea) for GC-TOF-MS analysis. For derivatization, 100 μl of the re-dissolved sample extract (10,000 ppm) was collected in 1.5 ml Eppendorf tubes and completely dried using a speed vacuum concentrator. The derivatization reaction involved oximation and silylation. For oximation, was 50 μl of methoxyamine hydrochloride (20 mg/ml in pyridine) added to the dried extract, and the mixture incubated for 90 min at 30°C. Next, silylation was performed by adding 50 μl of *N*-methyl-*N*-(trimethylsilyl) trifluoroacetamide to the mixture, followed by incubation for 30 min at 37°C. The final concentration of the derivatized sample extract was 10,000 ppm. All samples were filtered using a 0.2-μm PTFE filter (Chromdisc, Daegu, Korea) prior to instrument analyses. After injecting 1 μl into the GC-TOF-MS instrument in splitless mode, GC-TOF-MS analysis was performed using an Agilent 7890A GC system (Agilent Technologies, Palo Alto, CA, United States) coupled with an Agilent 7,693 autosampler (Agilent Technologies) and Pegasus HT TOF-MS (LECO Corp., St. Joseph, MI, United States). The chromatographic separation was conducted by an Rtx-5MS column (30 m × 0.25 mm, 0.25 μm particle size; Restek Corp., St. Joseph, MI, United States) with a helium as carrier gas. The analytical methods and operation parameters were as described in our previous study (Lee et al., 2016). Sample analysis was performed for three biological replicates and two analytical replicates.

#### Ultrahigh-Performance Liquid Chromatography-Linear Trap Quadrupole-Orbitrap-Tandem Mass Spectrometry Analysis for Secondary Metabolites

The dried extracts were re-dissolved in 80% MeOH (10,000 ppm) and filtered using a 0.2-μm PTFE filter (Chromdisc, Daegu, Korea) for UHPLC-LTQ-Orbitrap-MS/MS analysis. A UHPLC system was equipped with a Vanquish binary pump H system (Thermo Fisher Scientific, Waltham, MA, United States) coupled with an autosampler and column compartment. Chromatographic separation was performed using a Phenomenex KINETEX® C18 column (100 mm × 2.1 mm, 1.7 μm particle size; Torrance, CA, United States) using a mobile phase consisting of 0.1% (v/v) formic acid in water (solvent A) and 0.1% (v/v) formic acid in acetonitrile (solvent B). The gradient condition was designed as follows: 0–1 min, 5% B; 1–10 min, 5–100% B; 10–11 min, 100% B; 11–14 min, 100–5% B. The flow rate, injection volume, column temperature were set at 0.3 ml/min, 5 μl, and 40°C, respectively. Mass spectra were recorded in the full-spectrum mode covering 100–1,000 m/z using an Orbitrap Velos Pro^™^ system consisting of an ion trap mass spectrometer (Thermo Fisher Scientific) paired with a detector. Mass spectrometry detection designed as follows: capillary temperature, 350°C; and capillary voltage, 2.5 kV in negative mode and 3.7 kV in positive mode. The analytical methods and operation parameters were adopted from a previous study with some modifications (Mun et al., 2021). Sample analysis was performed for three biological replicates and two analytical replicates.

#### Headspace-Solid Phase Microextraction Gas Chromatography Time-of-Flight Mass Spectrometry Analysis for Volatile Organic Compounds (VOCs)

To extract VOCs from cucumber samples, 5 g of fresh cucumber was ground using liquid nitrogen. The homogenized sample was transferred into a 20 ml SPME glass vial (20 ml) and filled with 2 μl of solution (20% NaCl/Octanal (98:2, v/v)). For volatile collection, headspace-solid phase microextraction (HS-SPME) of the VOCs was performed using 50/30 μm divinylbenzene/carboxen^™^/polydimethylsiloxane (DVB-CAR-PDMS) StableFlex^™^ fiber (Sigma-Aldrich, St. Louis, MO, United States). The SPME fiber, preheated at 270°C for 1 min, was injected into the SPME vial and exposed to the headspace for 20 min at 60°C. Then, the fiber was introduced into the injector port of a GC–MS instrument (7890A GC-5975C MSD; Agilent) equipped with a DB-FFAP column (30 m × 0.25 mm, 0.25 μm film; J&W Scientific, Folsom, CA, United States). The injector (splitless mode) temperature was set at 250°C and extraction of VOCs was performed by exposing the SPME fiber to the headspace of sample supernatants for 30 min at 37°C. The oven temperature was initially set at 50°C for 2 min and increased to 300°C at a rate of 10°C min^−1^. After reaching 300°C, the temperature was held for 3 min. The temperature of the transfer line was set 240°C. After the extraction, fiber was removed from holder and desorbed at the GC port for 1 min at 270°C. The analytical method for VOCs was followed by Singh and Lee (2018) with some modifications. The sample analysis was performed using two analytical replicates.

### Data Processing and Multivariate Statistical Analysis

The GC-TOF-MS and HS-SPME-GC-TOF-MS raw data files were converted into NetCDF (*.cdf) format using the LECO ChromaTOF software (version 4.44). In addition, the UHPLC-LTQ-orbitrap-MS/MS raw data files were converted to NetCDF (*.cdf) format using Thermo Xcalibur software (version 2.1; Thermo Fisher Scientific). After conversion, NetCDF files were processed using the metAlign software package for peak detection, retention time correction, and alignment. The processed data were then used for SIMCA-P + 12.0 (Umetrics, Umea, Sweden) for principal component analysis (PCA) and partial least squares-discriminant analysis (PLS-DA). To select the significantly different metabolites among the samples, variable importance in the projection (VIP > 0.7) values based on a partial least squares-discriminant analysis (PLS-DA) score plot were applied. In addition, the significance test (*p* < 0.05) between biological and analytical replicates was performed by analysis of variance (ANOVA) and Duncan’s multiple-range tests using PASW Statistics 18 software (SPSS, Inc., Chicago, IL, United States). Selected metabolites were tentatively identified by comparison with various data, including mass fragment patterns, retention times, and the mass spectra of data for standard compounds under the same conditions from published papers and commercial databases, such as the National Institutes of Standards and Technology (NIST) Library (version 2.0, 2011, FairCom, Gaithersburg, MD, United States), and Wiley 9. A correlation map was obtained using the PASW Statistics software (version 18.0; SPSS Inc., Chicago, IL, United States).

### RNA Extraction and Transcriptome Analysis

**Figure 2:**
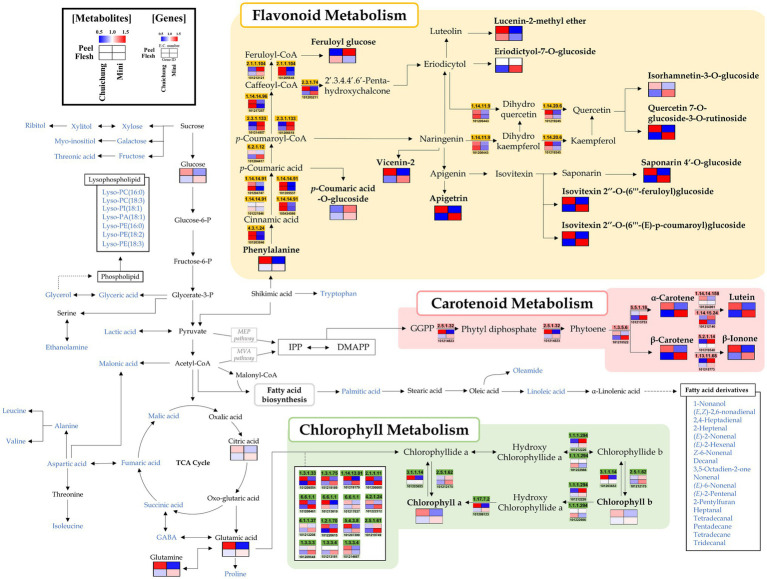
Schematic representation of the flavonoid, carotenoid, and chlorophyll metabolic pathway with metabolite and related gene expression levels in the Chuichung and Mini cultivars. The pathway was modified from the Kyoto Encyclopedia of Genes and Genomes (KEGG) database (http://www.genome.jp/kegg/). The colored squares (blue-to-red) represent the relative abundance. The blue characters represent metabolites detected through the mass spectrometry, and the relative abundance of these metabolites is shown in Figures 4, 5.

For RNA isolation, fresh cucumber samples were homogenized in liquid nitrogen using a mortar and pestle. The homogenized sample was transferred into Eppendorf tubes filled with TRI reagent solution and stored at −80°C until RNA purification. Library preparation and RNA sequencing were performed using the Illumina Hiseq4000 platform by Macrogen (Seoul, Korea; http://www.macrogen.com; accessed December 25, 2021). Prior to RNA sequencing, a 2,100 Bioanalyzer (Agilent, Böblingen, Germany) was used to determine the total RNA quality and amount separated from the samples. For library preparation, we confirmed that all samples had RNA integrity ≥7.5. Library construction was performed by using the TruSeq Stranded mRNA LT sample prep Kit (Illumina, San Diego, CA, United States) by Macrogen (Seoul, Korea). Raw data were trimmed by filtering according to the following criteria: unpaired reads, empty nucleotides, adaptor-only nucleotides, short reads (<36 bp), and low-quality nucleotides (Q-value: Phred quality value ≤20). Processed reads were aligned to reference genome data (GCF_000004075.3_Cucumber_9930_V3) using HISAT2 (version 2.1.0). After alignment, aligned reads were assembled into transcripts using StringTie (version 2.1.3b). The raw and trimmed data statistics were presented in supplementary table (Supplementary Tables S7, S8). Mapped data statistics summarized in Supplementary Table S9. The expression level of each transcript was normalized to the values of fragments per kilobase of exon per million fragments mapped (FPKM) and log2-transformed for quantile normalization. In addition, in the statistical analysis prior to DEG analysis, genes with a count value of 0 in at least one sample were excluded from the analysis. Therefore, the analysis was conducted on 16,816 genes of the total 23,995 genes excluding 7,179 (Supplementary Figure S7). DEGs in each sample were selected as *p* ≤ 0.05, log_2_ fold change values (FC) ≥2. The count of up and downregulation genes that present significantly differences based on fold change and value of *p* for each combination is summarized in Supplementary Figure S8. In this study, DEGs associated with flavonoid, carotenoid, and chlorophyll metabolic pathways were selectively identified and included in Figure 2. In addition, to compare the gene expression associated with the antioxidant activity of cucumber fruit, the main focus was the DEG of the Chuichung and Mini, which had the largest difference in activity in this study. RNA extraction and transcriptome analysis were performed as described by Son et al. (2019) with some modifications. We have submitted the raw sequence data to NCBI (BioProject: PRJNA817515).

## Results

### Difference in Morphological Features, Level of Bioactive Secondary Metabolites, and Antioxidant Activity in Three Cucumber Cultivars

To evaluate cucumber fruit quality, we examined the morphological characteristics, content of total phenolic and flavonoid compounds, carotenoids and chlorophylls, and antioxidant activities of three different cultivars. Figure 1 shows photographs of the three cucumber cultivars analyzed in this study. The morphological characteristics of cucumbers are listed in Table 1. The weight, length, and width of the cucumber fruit were measured, and these varied depending on the cultivar. These data indicated that cucumber fruit have various morphological characteristics based on their cultivar.

**Table 1:** Data on the morphological characterization of the three cucumber cultivars.

**No.**	**Cultivars**	**Abbreviation**	**Morphological characterization**			
			**Weight (g)**		**Length** **(cm)**	**Width** **(cm)**
			**Peel**	**Flesh**		
1	Chuichung	C	28.46 ± 0.50^a^	222.79 ± 2.33^a^	29.9 ± 0.79^a^	3.86 ± 0.15^a^
2	White Dadagi	W	30.41 ± 1.65^a^	212.42 ± 20.02^a^	24.2 ± 0.26^b^	3.73 ± 0.21^a^
3	Mini	M	10.30 ± 0.79^b^	50.51 ± 6.23^b^	11.0 ± 0.26^c^	2.73 ± 0.32^b^

The different alphabets (a, b, c) within the morphological characterization columns indicate values that are significantly distinguished according to the Duncan multiple-range test at value of *p* < 0.05.

**Figure 3:**
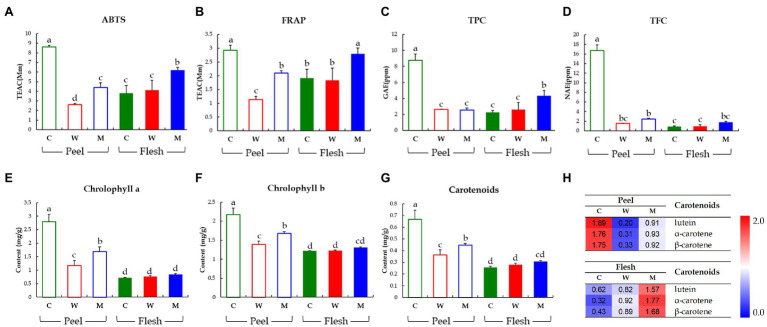
Results of antioxidant activities including ABTS assay (**A**) and FRAP assay (**B**), TPCs (**C**), TFCs (**D**), chlorophylls contents (**E, F**), and carotenoid analysis (**G, H**). Different letters in the bar graph indicate significant difference by ANOVA followed by Duncan’s multiple-range test (*p* < 0.05). ABTS, 2,2′-azino-bis (3-ethylbenzothiazoline-6-sulfonic acid); FRAP, ferric reducing antioxidant power; TEAC, trolox equivalent antioxidant capacity; GAE, gallic acid equivalent concentration; NAE, naringin equivalent concentration; C, Chuichung; W, White Dadagi; M, Mini.

To compare the bioactivities and bioactive secondary metabolite levels of the three cucumbers, we analyzed their antioxidant activities (ABTS and FRAP), TPC, TFC, and carotenoids and chlorophylls contents (Figure 3). In the peel, the antioxidant levels determined by ABTS and FRAP assays were significantly higher in Chuichung than in the others. In contrast, in the flesh, antioxidant activities were highest in Mini (Figures 3A,B). TPC and TFC were highest in the fruit peel of Chuichung and fruit flesh of Mini (Figures 3C,D). Chlorophylls and carotenoids contents also showed the same tendency as antioxidant activities (Figure 3E–G). We performed LC-DAD to confirm the relative abundance of each carotenoid compound, including lutein, α-carotene, and β-carotene. Lutein, α-carotene, and β-carotene were the highest in Chuichung peel. However, in the flesh, these compounds were the highest in Mini (Figure 3H).

Collectively, we observed that the levels of antioxidant activities and bioactive compound contents (carotenoids and chlorophylls) of cucumber fruit types showed different patterns in the peel and flesh. The content of carotenoids and chlorophylls, TFC, TPC, and antioxidant activity levels were the highest in the fruit peel of Chuichung and fruit flesh of Mini. All of these were confirmed to be statistically significant.

### Comparative Evaluation of Different Cucumber Cultivars Based On Metabolite Profiles

**Figure 4:**
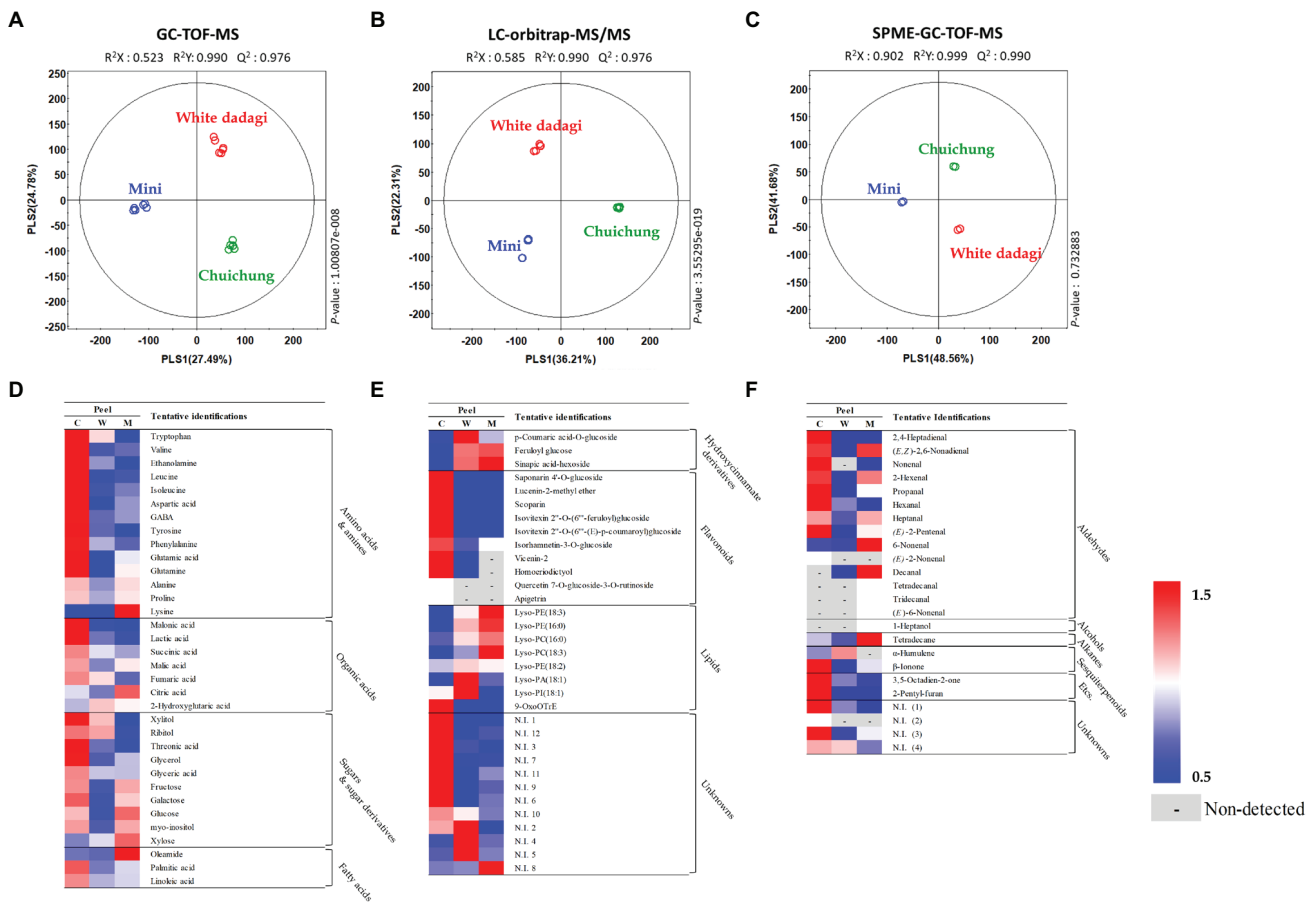
Partial least squares-discriminant analysis (PLS-DA; **A–C**) score plots for metabolites in three different cucumber peel based on GC-TOF-MS **(A)**, UHPLC-LTQ-Orbitrap-MS/MS **(B)**, and HS-SPME-GC-TOF-MS **(C)** dataset. Heat map analysis of different cucumber peel based on GC-TOF-MS **(D)**, UHPLC-LTQ-Orbitrap-ESI-MS/MS **(E)**, and HS-SPME-GC-TOF-MS **(F)** data. Heat map representation of the relative abundance of significantly discriminant metabolites (VIP > 0.7, *p* < 0.05) based on PLS-DA model. Peel of Chuichung (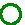), White Dadagi (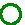), and Mini (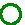) samples. GC-TOF-MS, gas chromatography time-of-flight mass spectrometry; UHPLC-LTQ-Orbitrap-MS/MS, ultrahigh-performance liquid chromatography–linear trap quadrupole-orbitrap–tandem mass spectrometry; HS-SPME-GC-TOF-MS, headspace-solid phase microextraction gas chromatography time-of-flight mass spectrometry; VIP, variable importance projection; PLS-DA, partial least squares-discriminant analysis.

**Figure 5:**
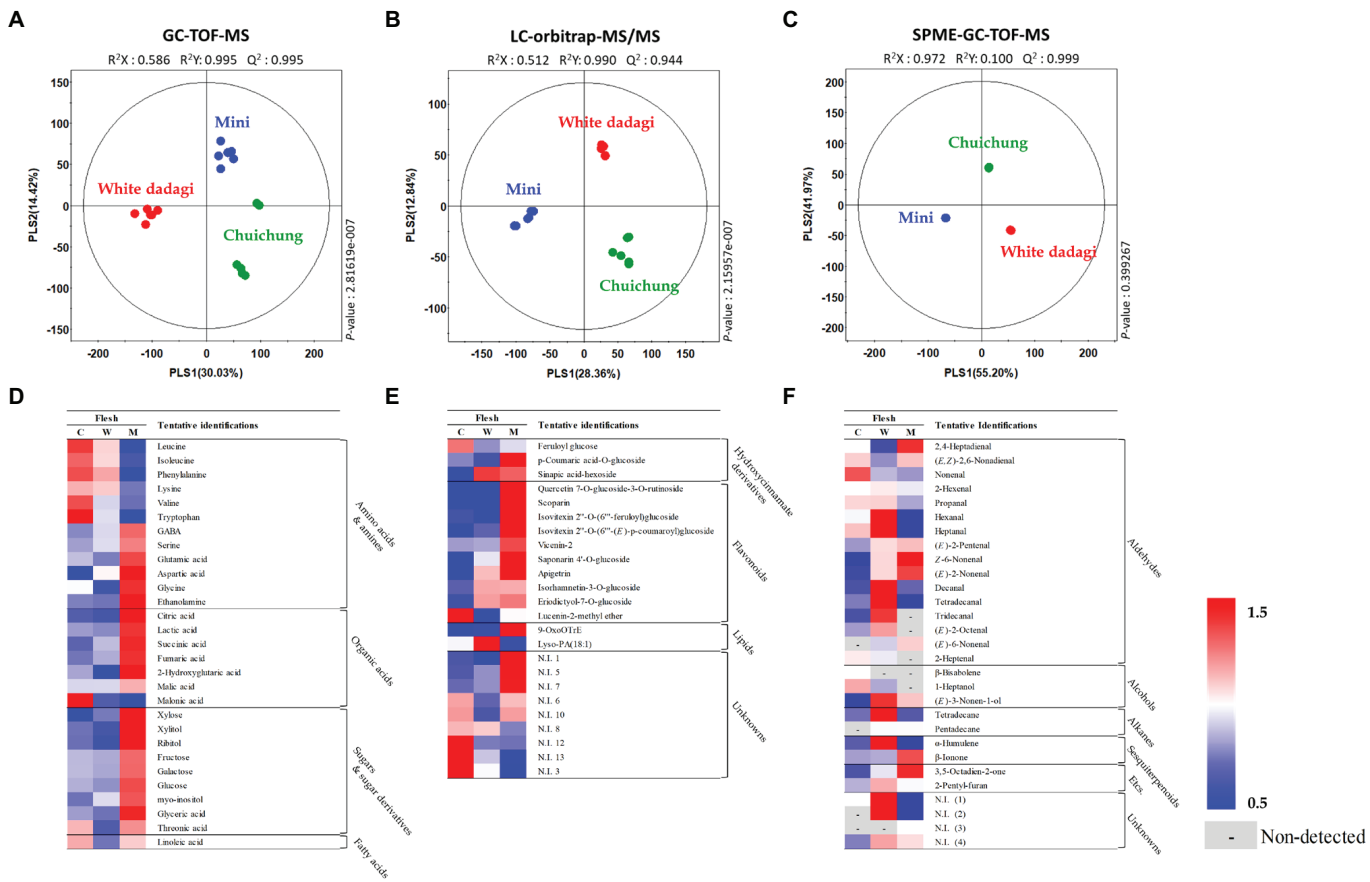
Partial least squares-discriminant analysis (PLS-DA; **A**–**C**) score plots for metabolites in three different cucumber flesh based on GC-TOF-MS (**A**), UHPLC-LTQ-Orbitrap-MS/MS (**B**), and HS-SPME-GC-TOF-MS (**C**) data set. Heat map analysis of different cucumber flesh based on GC-TOF-MS (**D**), UHPLC-LTQ-Orbitrap-ESI-MS/MS (**E**), and HS-SPME-GC-TOF-MS (**F**) data. Heat map representation of the relative abundance of significantly discriminant metabolites (VIP > 0.7, *p* < 0.05) based on PLS-DA model. Flesh of Chuichung (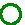), White Dadagi (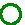), and Mini (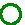) samples. GC-TOF-MS, gas chromatography time-of-flight mass spectrometry; UHPLC-LTQ-Orbitrap-MS/MS, ultrahigh-performance liquid chromatography–linear trap quadrupole-orbitrap–tandem mass spectrometry; HS-SPME-GC-TOF-MS, headspace-solid phase microextraction gas chromatography time-of-flight mass spectrometry; VIP, variable importance projection; PLS-DA, partial least squares-discriminant analysis.

To analyze the global metabolites in the three different cucumbers, metabolite profiling was performed using HS-SPME-GC-TOF-MS, GC-TOF-MS, and UHPLC-LTQ-Orbitrap-MS/MS. To evaluate significantly discriminated metabolites derived from cucumber, we performed multivariate analyses including PCA and PLS-DA. The results revealed that the three commercial cucumbers were distinguished from each other and clustered depending on their type (Supplementary Figures S1, S2). The PLS-DA score plot also showed a similar pattern to that of the PCA score plot (Figures 4A–C and Figures 5A–C). To investigate distinctive metabolites among the different types of cucumbers, we selected significantly discriminated metabolites based on the VIP value (>0.7) derived from the PLS-DA model and *p* < 0.05.

In the peel, the PLS-DA model derived from GC-TOF-MS, UHPLC-LTQ-Orbitrap-MS/MS, and HS-SPME-GC-TOF-MS datasets, three cucumber samples showed distinct patterns of PLS1 and PLS2 (Figures 4A–C). Notably, the PLS-DA model for UHPLC-LTQ-Orbitrap-MS/MS data showed that Chuichung was clearly distinguished from the others by PLS1 (36.21%), concurrent with the antioxidant activity (Figure 4B). A total of 80 distinguished metabolites were identified, of which 34, 21, and 25 metabolites were identified by GC-TOF-MS, UHPLC-LTQ-Orbitrap-MS/MS, and HS-SPME-GC-TOF-MS, respectively (Supplementary Tables S1–S3). These identified metabolites were categorized into the following metabolite classes: 14 amino acids, nine organic acids, eight sugar derivatives, 10 flavonoids, 11 lipids (three fatty acids, one fatty acid amide, and seven lysophospholipids), and 25 volatile compounds (16 aldehydes, three alcohols, two alkanes, two sesquiterpenoids, and two others).

In the flesh, the PLS-DA model for GC-TOF-MS, UHPLC-LTQ-Orbitrap-MS/MS, and HS-SPME-GC-TOF-MS datasets also displayed clearly distinguished patterns among the three different cucumbers by PLS1 and PLS2, respectively (Figures 5A–C). Contrary to the peel, the PLS-DA model for UHPLC-LTQ-Orbitrap-MS/MS data showed that Mini was clearly distinct from the others by PLS1 (28.36%; Figures 5B). A total of 69 distinguished metabolites were identified, of which 29, 15, and 25 metabolites were identified by GC-TOF-MS, UHPLC-LTQ-Orbitrap-MS/MS, and HS-SPME-GC-TOF-MS, respectively (Supplementary Tables S4–S6). These identified metabolites included 12 amino acids, nine organic acids, seven sugar derivatives, 10 flavonoids, three lipids (two fatty acids and one lysophospholipids), and 25 volatile compounds (16 aldehydes, three alcohols, two alkanes, two sesquiterpenoids, and two others).

For the visualization of significantly different metabolites, all metabolites were displayed on a heat scale (Figures 4D–F). Each column is expressed as a fold change calculated from the average peak area of each type of cucumber. In the peel, the levels of primary metabolites, such as amino acids (tryptophan, valine, ethanolamine, leucine, isoleucine, aspartic acid, GABA, tyrosine, phenylalanine, glutamic acid, and glutamine), organic acids (malonic acid, lactic acid, succinic acid, malic acid, and fumaric acid), and sugar derivatives (xylitol, ribitol, threonic acid, glycerol, glyceric acid, galactose, and myo-inositol), were relatively higher in Chuichung than in other cucumbers (Figure 4D). The levels of secondary metabolites were showed different metabolite pattern according to cucumber cultivars. Most of flavonoid compounds (saponarin 4’-*O*-glucoside, vicenin-2, lucenin-2-methyl ether, scoparin, isovitexin 2″-*O*-(6″‘-feruloyl)glucoside, isovitexin 2″-O-(6″‘-(E)-p-coumaroyl) glucoside, isorhamnetin-3-O-glucoside, and homoeriodictyol) were relatively higher in Chuichung than in other cucumbers. In particular, quercetin 7-O-glucoside-3-O-rutinoside and apigetrin were detected in only Chuichung. Hydroxycinnamate derivatives and lipids were observed the lowest level in Chuichung (Figure 4E). Volatile compounds also showed significantly different patterns according to the fruit cultivars. In this study, 24 volatile compounds were tentatively identified as a compound have significantly differences among the peel of the three cucumber cultivars. Fourteen of 24 volatile compounds belonged to the aldehyde. In particular, aldehyde including 2,4-heptadienal, nonenal, propanal, hexanal, and *(E)*-2-pentenal showed clearly higher level in Chuichung than other cultivars. However, 6-nonenal and decanal showed higher level in Mini than others. Specifically, *(E)*-2-nonenal was only detected in Chuichung and tridecanal, tetradecanal, and *(E)*-6-nonenal were detected only in Mini (Figure 4F).

In the flesh, the levels of the most of primary metabolites, such as some amino acids (GABA, serine, glutamic acid, aspartic acid, glycine, and ethanolamine), organic acids (citric acid, lactic acid, succinic acid, malic acid, fumaric acid, and 2-hydroxyglutaric acid), and sugar derivatives (xylose, xylitol, ribitol, fructose, galactose, glucose, myo-inositol, glyceric acid, and threonic acid), were observed that higher abundance in Mini than in other cucumbers (Figure 5D). The level of secondary metabolite also showed different patterns by cultivars. Most of flavonoid compounds including quercetin 7-O-glucoside-3-O-rutinoside, scoparin, isovitexin 2″-O-(6″‘-feruloyl)glucoside, isovitexin 2″-O-(6″‘-*(E)*-*p*-coumaroyl)glucoside, vicenin-2, saponarin 4’-O-glucoside, and apigetrin were showed that relatively higher level in Mini than in other cucumbers (Figure 5E). Volatile compounds showed significantly different patterns according to the fruit cultivars. In this study, 29 volatile compounds were tentatively identified as a compound have significantly differences among the flesh of the three cucumber cultivars. Among VOCs, the most detected aldehyde, such as 2,4-heptadienal, *(E,Z)*-2,6-nonadienal, *(E)*-2-pentenal, *(Z)*-6-nonenal, *(E)*-2-nonenal, and *(E)*-6-nonenal, were showed the highest level in Mini. On the other hand, 2-hexenal, hexanal, heptanal, decanal, tetradecanal, tridecanal, and *(E)*-2-octenal were observed that higher level in White Dadagi than others. Nonenal and 2-heptenal were represented the highest pattern in Chuichung (Figure 5F).

### Integrated Pathway Mapping for Discriminant Metabolites and DEGs In Chuichung and Mini Cultivars

To identify the compounds that contribute to antioxidant activity, we conducted a correlation analysis between metabolites and activities with Chuichung and Mini, which showed the most characteristic patterns in each part (Supplementary Figure S3). In both peel and flesh, apigenin and quercetin derivatives, chlorophyll a, chlorophyll b, lutein, α-carotene, and β-carotene were significantly positively correlated with the antioxidant activity.

Moreover, we performed a comprehensive metabolic pathway analysis through an metabolomics and DEGs analysis data to understand the detailed biosynthesis of positively correlated secondary metabolites with antioxidant activity (Figure 2).

According to pathway analysis, α-carotene, β-carotene, and lutein presented high relative abundance in Chuichung peel, whereas in flesh, they were higher on Mini. Moreover, β-ionone, a carotenoid-derived volatile compound, exhibited the same pattern. The expression patterns of 15-*cis*-pytoene synthase (EC 2.5.1.32) and ζ-carotene desaturase (EC 1.3.5.6), which are involved in the synthesis of precursors of these carotenoids, were consistent with the measured carotenoids contents. The chlorophyll a and chlorophyll b contents showed the same tendency as carotenoids. Most genes related to chlorophyll synthesis and regulation, such as protochlorophyllide reductase (EC 1.3.1.33), magnesium protoporphyrin IX monomethyl ester (oxidative) cyclase (EC 1.14.13.81), magnesium protoporphyrin O-methyltransferase (EC 2.1.1.11), magnesium chelatase subunit D (EC 6.6.1.1), glutamyl-tRNA reductase (EC 1.2.1.70), glutamate-1-semialdehyde 2,1-aminomutase (EC 5.4.3.8), heme oxygenase (biliverdin-producing, ferredoxin; EC 1.14.15.20), porphobilinogen synthase (EC 4.2.1.24), uroporphyrinogen decarboxylase (EC 4.1.1.37), chlorophyllase (EC 3.1.1.14), and 7-hydroxymethyl chlorophyll a reductase (EC 1.17.7.2), showed higher expression levels in Chuichung peel and Mini flesh. Flavonoids, such as apigenin derivatives (apigetrin, saponarin 4’-*O*-glucoside, isovitexin 2″-*O*-(feruloyl)-glucoside, and isovitexin 2″-*O*-(6″-(*E*)-*p*-coumaroyl)-glucoside) and quercetin derivatives (isorhamnetin-3-*O*-glucoside and quercetin-7-*O*-glucoside-3-*O*-rutinoside), were observed at a relatively higher abundance in Chuichung peel and in Mini flesh. Phenylalanine, a precursor of flavonoid biosynthesis, showed the same pattern. However, unlike carotenoid and chlorophyll metabolism, flavonoid-related genes were highly expressed in the peels of both cucumber cultivars.

## Discussion

Cucumber is consumed around the world for its flavor, important nutrients, and bioactive compounds. In addition, cucumber is also used in therapeutic medicine and cosmetic fields. Currently, various cultivars of cucumbers have been developed to appeal to consumers by enhancing fruit quality. The fruit quality of cucumbers is determined by various factors, such as morphological traits, flavors, and biological properties (Cuthbertson et al., 2012). However, few studies have comprehensively investigated the quality of various cultivars. In this study, we performed the comprehensive research to compare the fruit quality among different cucumber cultivars: Chuichung and White Dadagi, which are mainly consumed in Korea, and Mini, was recently developed in Korea.

### Comparison of Odor In Cucumber Fruit

In the peel, most alcohols (2,4-heptadienal, (*E,Z*)-2,6-nonadienal, nonenal, 2-hexenal, propanal, hexanal, heptanal, (*E*)-2-pentenal, and (*E*)-2-nonenal) were higher in Chuichung than in the others. In addition, the volatiles β-ionone, 3,5-octadien-2-one, and 2-pentyl-furan showed higher patterns in Chuichung than in the others. White Dadagi showed the lowest relative abundance of VOCs, except for α-humulene (Figure 4F). In the flesh, most alcohols (2-hexenal, propanal, hexanal, heptanal, (*E*)-2-pentenal, (*E*)-2-nonenal, decanal, tetradecanal, tridecanal, and (*E*)-2-octanal), 2-pentyl-furan, and α-humulene were present at higher levels in White Dadagi than in the other groups (Figure 5F). In agreement with our results, similar classes of VOCs have been reported in cucumber (Chen et al., 2015; Wei et al., 2016). The volatiles 2,4-heptadienal, nonenal, 2-hexenal, and 3,5-octadien-2-one have been reported to be important contributors to green and fresh odors. Heptanal, (*E*)-2-pentenal, and 2-pentyl-furan are known to have fruity odors. In addition, β-ionone has a flower-like odor (Chen et al., 2015; Wei et al., 2016). Therefore, it can be inferred that Chuichung peel has an abundant and diverse odor compared to other cucumber cultivars; however, White Dadagi flesh has more varied types of odors than the other cultivars. Volatile (*E,Z*)-2,6-nonadienal, which is derived from the 9-hydroperoxides of linolenic acid, has been associated with a cucumber-like odor and has been reported as the major volatile component of cucumber aroma (Grosch and Schwarz, 1971). In both the peel and flesh, the relative proportions of (*E,Z*)-2,6-nonadienal were found to be comparatively higher in Chuichung and Mini than in White Dadagi (Figures 4F, 5F). Collectively, the characteristic odors of cucumber can be found in Chuichung and Mini.

### Comparison of Taste and Nutritional Compounds In Cucumber Fruit

Cucumber fruit mainly have sweet and bitter tastes. Previous studies have shown that the amino acid content determines various factors affecting the taste, flavor, nutrition, and palatability of fruit (Youn et al., 2007). The glycine and serine contents determine the sweetness of fruit, whereas valine, leucine, isoleucine, and phenylalanine confer a bitter taste (Keutgen and Pawelzik, 2008). In our study, in the peel, the sweet taste-related metabolites, such as glycine, serine, and sugar derivatives, and bitter taste-related metabolites, including valine, leucine, isoleucine, and phenylalanine, were higher in Chuichung (Figure 4D). In the flesh, some amino acids related to bitter taste were higher in Chuichung; however, compounds related to sweet taste were higher in Mini (Figure 5D).

Cucumber fruit also contain various nutritionally and functionally important secondary metabolites (Mukherjee et al., 2013). According to their research, flavonoids, carotenoids, and chlorophylls are important secondary metabolites of cucumber fruit.

Flavonoids are polyphenolic secondary metabolites that have been identified in many plants (Tadmor et al., 2010). Fruit and vegetable polyphenols are phenolics including coumarins, phenolic acids, like hydroxybenzoic acids (Sarker and Oba, 2020c) and hydroxycinnamic acids (Sarker, 2018), flavonoids, such as flavonols (Sarker and Oba, 2019), flavones (Sarker and Oba, 2020d), flavanols (Sarker and Oba, 2020b), flavanones (Sarker et al., 2020), isoflavones, anthocyanins, chalcones and non-flavonoids, such as tannins, lignans, and stilbenes, with strong radical quenching ability (Sarker and Oba, 2020e). In this study, we identified apigenin and quercetin derivatives. Apigenin and quercetin are flavones and flavanols, respectively, which are water-soluble pigments contained in vegetables and are generally reported to have a pale yellow color (Alihosseini and Sun, 2011; Awika, 2017). Flavonoids are considered antioxidants because they can remove free radicals, inhibit lipid oxidation, or chelate metal ions (Tripoli et al., 2007). The reason for the increased scavenging activity of flavonoids is mainly due to the C2−C3 double bond with the 4-oxo group of C-rings, but is also affected by the number of hydroxyl groups on the B-rings (Borges Bubols et al., 2013).

Carotenoids are tetraterpenoids, namely, α-carotene, β-carotene, (Sarker and Oba, 2021), xanthophylls like neoxanthin, violaxanthin, zeaxanthin, lutein (Sarker and Oba, 2020a), lycopene, etc. having strong antioxidants capacity (Sarker and Oba, 2018a,b, 2020e) which are responsible for yellow, orange-red, and red colors (Miller et al., 2014; Aadil et al., 2019). These compounds are likely to be involved in the prevention or protection of severe human health disorders, such as heart disease, cancer, and macular degeneration (Agarwal and Rao, 2000; Fiedor and Burda, 2014). Carotenoids are composed of a 40-carbon skeleton of isoprene units covalently linked together, forming multiple conjugated double bonds. The biggest feature of this structure is the long series of conjugated double bonds that form the central part of the molecule. It gives them a compound shape, chemical reactivity, and light-absorbing properties (Miller et al., 2014. Carotenoids with these structural characteristics are known to be effective antioxidants because of their ability to remove free radicals (Pérez-gálvez et al., 2020).

Chlorophylls are tetrapyrrole metabolites that indicates green color present in plants and serves to convert light energy into chemical energy in plants through a process known as photosynthesis (Ares et al., 2021). Chlorophylls have a characteristic central structure similar to the heme structure of hemoglobin constituting blood (Pérez-gálvez et al., 2020). Therefore, chlorophyll supplies oxygen to the blood and helps detoxify human organisms. Other health-promoting effects caused by its antioxidant and anti-inflammatory activities have also been reported in previous studies (Perez-Galvez et al., 2017).

These metabolites are crucial for pigments and antioxidant compounds in cucumber (Lanfer-Marquez et al., 2005; Złotek et al., 2014; Lai et al., 2015; Wang et al., 2020). Previous studies have reported that the contents of chlorophylls, carotenoids, and flavonoids are much higher in dark-green cucumbers than in light-green cucumbers (Wang et al., 2020). In addition, Mun et al. (2021) reported that the levels of flavonoids, carotenoids, and antioxidant activity were relatively high in fruit of cultivars that were smaller than the conventional cultivars of general size.

In our study, after separating the skin and flesh, the relative abundance levels of these metabolites and antioxidant activity were analyzed according to cultivar. Similarly, these compounds were relatively higher in the peel of Chuichung, which had the darkest color (Figures 3E–H, 4E). In the flesh, although the color difference of each cucumber was not observed as clearly as in the peel, the contents of chlorophyll b and carotenoids were significantly higher in Mini, which had a relatively darker color (Figures 3F–H, 5E). Therefore, we found that the compounds involved in the taste and nutrition of fruit differed depending on the cultivar and that secondary metabolites associated with various activities showed relatively high levels in Chuichung peel and Mini flesh. This result was consistent with our results from the antioxidant activity assay (Figures 3A,B). Comprehensively considering our results, based on our metabolite profiling and functional studies, we assumed that the color of cucumbers was associated with antioxidant activity. The association between fruit color and antioxidant activity has also been reported in previous studies (Hua et al., 2018; Shi et al., 2018).

### Metabolism of Bioactive Compounds In Cucumber Fruit

Antioxidant activity prevents and suppresses various diseases and aging by removing free radicals (Masaki, 2010; Song et al., 2010; Fiedor and Burda, 2014). Thus, cucumber fruit can be used in various food and cosmetic industries. Therefore, this is an important factor in evaluating cucumber fruit quality. In the present study, the patterns of antioxidant activity showed contradictory phenomena in the peel and flesh. Therefore, we conducted a correlation analysis to understand the different patterns of bioactivity in the peel and flesh and performed a correlation analysis between secondary metabolites and antioxidant activity. According to our results, we estimated that flavonoids, chlorophylls, and carotenoids had a significantly positive correlation with antioxidant activity in both the peel and flesh (Supplementary Figure S3).

We performed an integrated metabolic pathway analysis to understand the biosynthesis mechanism of bioactive compounds (Figure 2). Based on pathway analysis, we observed differences in the metabolism of bioactive compounds between Chuichung and Mini, which were the most distinguished in the results of UHPLC-LTQ-orbitrap-MS/MS analysis and antioxidant activity.

The mechanism of flavonoid synthesis and related genes have been described in previous studies (Dao et al., 2011; Zhang et al., 2020). Genes including phenylalanine ammonia lyase (*PAL*; EC 4.3.1.24), *trans*-cinnamate-4-monooxygenase (*C4H*; EC 1.14.14.91), four coumarate-CoA-ligases (*4CL*; EC 6.2.1.12), chalcone synthase (*CHS*; EC 2.3.1.74), naringenin, and 2-oxoglutarate 3-dioxygenase (*F3H*; EC 1.14.11.9) are known to play an important role in the flavonoid synthesis pathway (Dao et al., 2011; Zhang et al., 2020). *PAL*, *C4H*, *4CL*, and *CHS* catalyze the conversion of phenylalanine to naringenin chalcone. Subsequently, *chalcone isomerase (CHI*) catalyzes the conversion of naringenin chalcone to naringenin, which is then converted to dihydrokaempferol by *F3H* (Yu et al., 2021). Our study identified 14 DEGs involved in flavonoid metabolism. The 14 DEGs were displayed in the flavonoid pathway analysis (Supplementary Table S10; Figure 5). We found that *CHS* and *4CL* were upregulated in Chuichung peel and Mini flesh, and tentatively identified flavonoids, such as apigetrin, quercetin derivatives, and isovitexin derivatives, that were also upregulated in Chuichung peel and Mini flesh (Figure 2). This indicated that *CHS* was significantly more expressed in Chuichung peel and Mini flesh, which led to the upregulation of flavonoids. However, *C4H* was upregulated in Mini peel and Chuichung flesh, and *PAL* and *F3H* showed higher expression levels in Chuichung in both peel and flesh. Wang et al. (2020) reported that *CHS* and *4CL* play key roles in the synthesis of flavonoids and are significantly more expressed in dark-green cucumbers than in light-green cucumbers. Wang et al. (2020) also conducted research on genes that play a key role in the synthesis of flavonoids and reported that *CHS* and *4CL* are significantly more highly expressed in dark-green cucumbers than in light-green cucumbers. This result was consistent with our results. Therefore, in this study, it was found that various key genes regulating flavonoid synthesis mechanisms, including *PAL*, *C4H*, *4CL*, *CHS*, and *F3H*, were expressed differently in each type of cucumber.

The carotenoid synthesis mechanism has been previously described (Rodríguez-Concepción, 2010; Grassi et al., 2013). In addition, previous studies have shown that several genes, such as geranylgeranyl diphosphate synthase (*GGPPS*; EC 2.5.1.29), phytoene synthase (*PSY*; EC 2.5.1.32), and ζ-carotene desaturase (*ZDS*; EC 1.3.5.6), are involved in the carotenoid synthesis pathway (Rodríguez-Concepción, 2010; Grassi et al., 2013). Carotenoids are mainly biosynthesized from isopentenyl diphosphate (IPP) and dimethylallyl diphosphate (DMAPP) *via* the methylerythritol 4-phosphate (MEP) pathway in plastids (Rodríguez-Concepción, 2010; Grassi et al., 2013). Geranylgeranyl diphosphate (GGPP) was synthesized using *GGPPS*. Then, the two GGPP molecules start to condense to produce phytoene by *PSY*. Subsequently, phytoene desaturase (*PDS*; EC 1.3.5.5) and *ZDS* catalyze similar dehydrogenation reactions by introducing four double bonds in phytoene to form lycopene, which is a precursor of α-carotene, β-carotene, and lutein. In this study, 21 DEGs involved in carotenoid metabolism were identified. Among these, seven were selected to visualize the integrated carotenoid pathway analysis (Supplementary Table S10; Figure 2). The expression of DEGs in the synthesis of carotenoids, genes associated with phytoene and lycopene synthase, *PSY* and *ZDS*, were highly upregulated in Chuichung peel and Mini flesh. These results are consistent with the higher abundance of carotenoids in Chuichung peel and Mini flesh. β-ionone, a carotenoid-derived volatile compound, also showed the same pattern as the other carotenoid compounds (Figure 2). Therefore, it seems that the gene expression of *PSY* and *ZDS* is a key mechanism regulating the production of carotenoids, and these key genes may accelerate carotenoid synthesis in Chuichung peel and Mini flesh. It was reported by Rodrigo et al. (2004) that increased *PSY* and *ZDS* gene expression plays an important role in enhancing the production of carotenoids in this pathway.

Genes involved in chlorophyll synthesis have been found in plant leaf and fruit, and chlorophyll synthesis mechanisms have been well studied (Lai et al., 2015; Wen et al., 2015). Genes including glutamyl-tRNA reductase 2 (*HemA*; EC 1.2.1.70), delta-aminolevulinic acid dehydratase (*HemB*; EC 4.2.1.24), uroporphyrinogen decarboxylase (*HemE*; EC 4.1.1.37), magnesium chelatase subunit chlH (*chlH*; EC 6.6.1.1), magnesium protoporphyrin IX methyltransferase (*chlM*; EC 2.1.1.11), chlorophyll(ide) b reductase (*NOL*; EC 1.1.1.294), and protochlorophyllide oxidoreductase (*por*; EC 1.3.1.33) are known to play important roles in the chlorophyll biosynthesis pathway (Tanaka and Tanaka, 2006, 2007). *HemA* is an enzyme that participates in chlorophyll biosynthesis in the plastids. It catalyzes the biosynthesis of 5-aminolevulinic acid from glutamyl-tRNA (Eckhardt et al., 2004). ChlH catalyzes the conversion of protoporphyrin IX to Mg-protoporphyrin IX. Magnesium protoporphyrin IX monomethyl ester formation catalyzes magnesium protoporphyrin IX in the chlorophyll synthesis pathway by *chlM* (Wang et al., 2017). *Por* is an important enzyme that catalyzes protochlorophyllide to generate chlorophyllide, which is a critical intermediate step in the conversion of chlorophyll (Kwon et al., 2017). In this study, 31 DEGs associated with chlorophyll metabolism were identified. Twenty of the 31 DEGs were displayed in the chlorophyll pathway analysis (Supplementary Table S10; Figure 2). With regards to the expression of DEGs in the chlorophyll synthesis pathway, most of the genes, including *HemA*, *HemB*, *HemE*, *chlH*, *chlM*, *chlE*, *CLH, DVR*, and *por*, were upregulated in Chuichung peel and Mini flesh. These results are consistent with the higher abundance of chlorophyll a and chlorophyll b in Chuicung peel and Mini flesh (Figure 2). This suggests that the expression of various key genes, including *HemA*, *HemB*, *HemE*, *chlH*, *chlM*, *chlE*, *CLH*, *DVR*, and *por*, is crucial for regulating the production of chlorophylls, and these genes may accelerate chlorophyll synthesis in Chuichung peel and Mini flesh. A previous study (Tanaka and Tanaka, 2007) found that the expression levels of the aforementioned genes play an important role in improving chlorophyll production in the pathway mechanism.

The present study evaluated various genes involved in flavonoid, carotenoid, and chlorophyll metabolisms. In the flavonoid pathway analysis, we proposed that *CHS* is a key gene for the regulation of flavonoid compounds. Analysis of the carotenoid and chlorophyll pathways confirmed that the expression patterns of genes involved in the synthesis of precursors corresponded to metabolite patterns. Therefore, from the results of this study, we could infer that the synthesis of a phytoene, a precursor of carotenoids, and chlorophyllide a, a precursor of chlorophylls, occurs differently depending on the cultivar and part of cucumbers, which leads to differences in fruit activity. This biosynthetic pathway analysis provides insight into disparities in metabolism distribution.

## Conclusion

This study demonstrates that the metabolomics approach is effective for investigating different cultivars that have various quality parameters. We conducted metabolomics study to evaluate three different cucumber cultivars (Chuichung, White Dadagi, and Mini) and their parts (peel and flesh). Although amino acids, sugars, flavonoids, carotenoids, and chlorophylls were found to be upregulated in Mini flesh, in the case of peel, these were expressed at higher levels in Chuichung. In addition, antioxidant activity was high in both Chuichung peel and Mini flesh. To interpret the different tendencies of bioactivity in their parts, we performed a comprehensive pathway analysis about the flavonoids, carotenoids, and chlorophylls, which had significantly positive correlation with antioxidant activity, using an integrated metabolomics and transcriptomics approach. The expression levels of flavonoid-related genes (*CHS* and *4CL*), carotenoid-related genes (*PSY* and *ZDS*), and chlorophyll-related genes (*HemA*, *HemB*, *HemE*, *chlH*, *chlM*, *chlE*, *CLH*, *DVR*, and *por*) were indicated the same tendency with the metabolome analysis data. As a result, we could estimate that flavonoid, carotenoid, and chlorophyll metabolism were upregulated in Chuichung peel and Mini flesh. These findings provide an insight of various commercial cucumbers and help explore valuable metabolites and genes associated with improving cucumber fruit quality. Also, these results provide valuable information of different characteristics in various cucumber cultivars, which can be considered while purchasing and consuming the cucumber fruit.

## Data Availability Statement

The datasets presented in this study can be found in online repositories. The names of the repository/repositories and accession number(s) can be found at: National Center for Biotechnology Information (NCBI) BioProject database under accession number PRJNA817515.

## Author Contributions

HJ, SS, and CL conceived of and designed the study. HJ collected the phenotypic data, analyzed the data, and wrote the manuscript. SS assisted with data analysis and reviewed the manuscript. All authors have contributed to the manuscript and approved the submitted version.

## Funding

This paper resulted from the Konkuk University research support program.

## Conflict of Interest

The authors declare that the research was conducted in the absence of any commercial or financial relationships that could be construed as a potential conflict of interest.

## Publisher’s Note

All claims expressed in this article are solely those of the authors and do not necessarily represent those of their affiliated organizations, or those of the publisher, the editors and the reviewers. Any product that may be evaluated in this article, or claim that may be made by its manufacturer, is not guaranteed or endorsed by the publisher.
