# Supplementary material for: Comparison of Metabolome and Functional Properties of Three Korean Cucumber Cultivars
Source: Front Plant Sci. 2022 Apr 15;13:882120. doi: 10.3389/fpls.2022.882120 (PMC9051474; doi:10.3389/fpls.2022.882120)
Supplement: Supplementary file 2 [file Data_Sheet_2.docx]

Supplementary Material

**
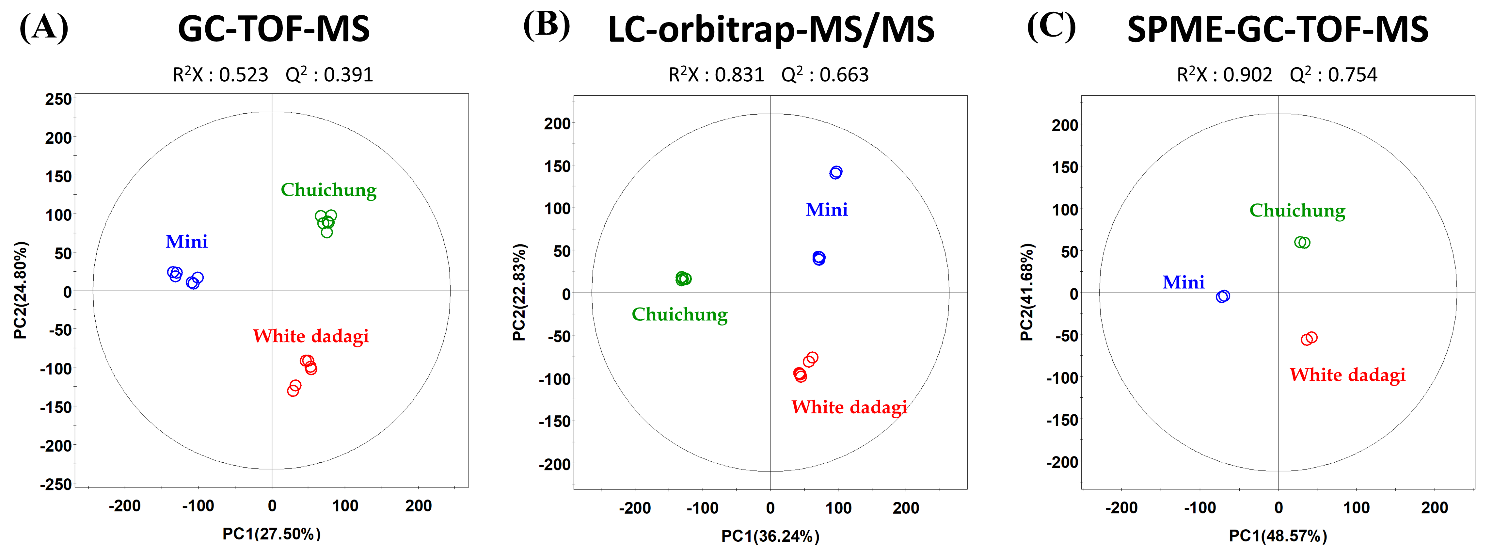
Figure S1.** Principal component analysis (PCA) score plots for in three different cucumber peel based on GC-TOF-MS (A), UHPLC-LTQ-Orbitrap-MS/MS (B) and HS-SPME-GC-TOF-MS (C) data set. Chuichung (○), White dadagi (○), and Mini (○) samples.

**
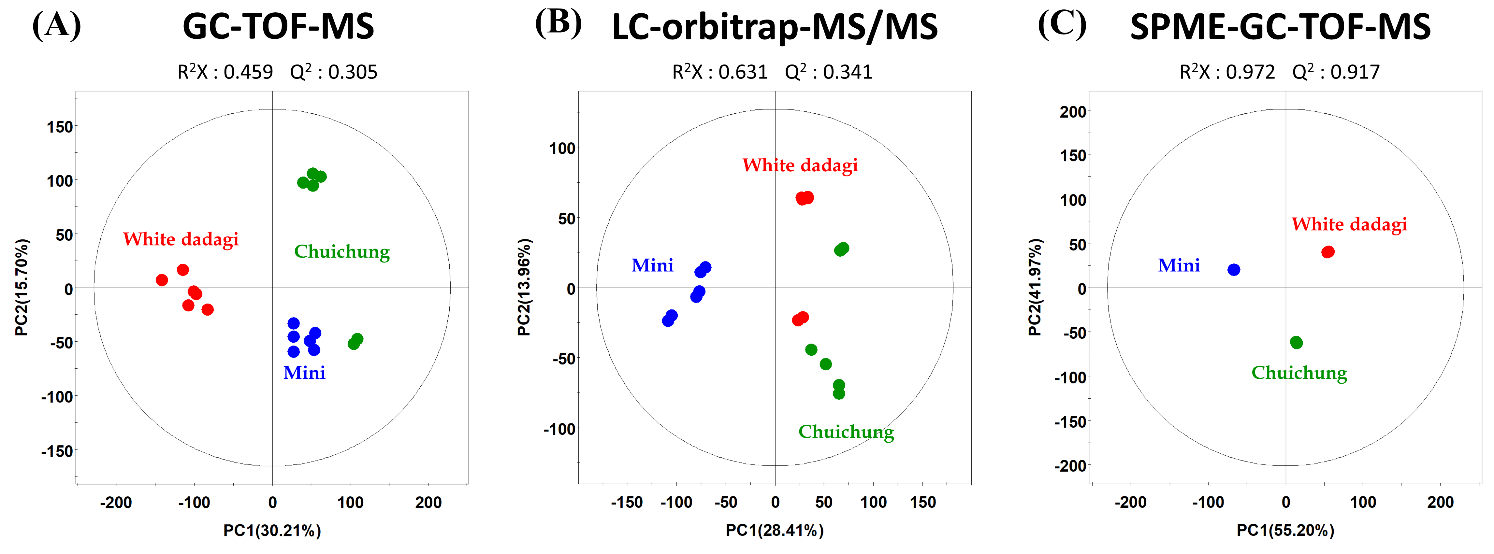
Figure S2.** Principal component analysis (PCA) score plots for in three different cucumber flesh based on GC-TOF-MS (A), UHPLC-LTQ-Orbitrap-MS/MS (B) and HS-SPME-GC-TOF-MS (C) data set. Chuichung (**●**), White dadagi (**●**), and Mini (**●**) samples.

**
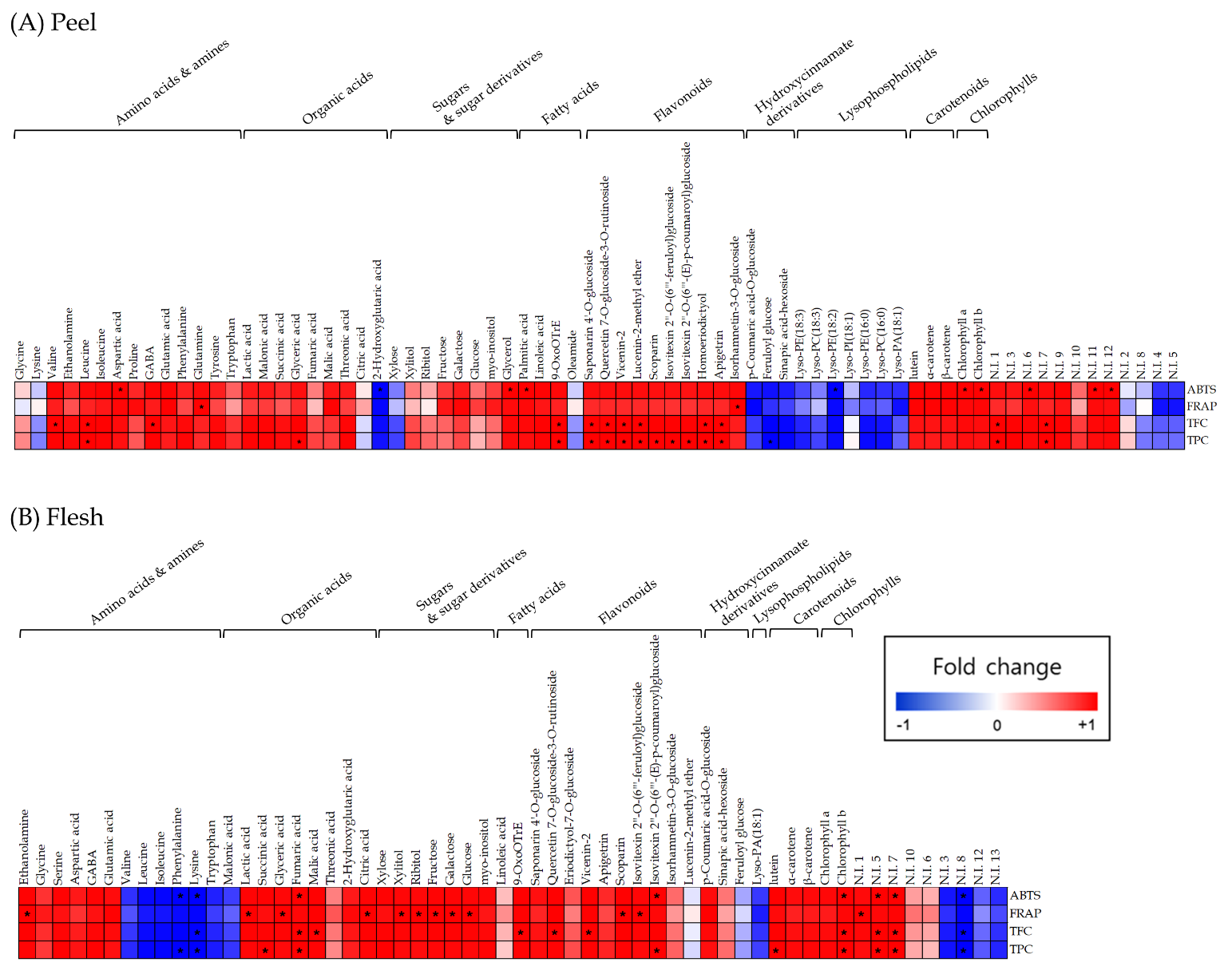
**

**Figure S3.** Correlation map between antioxidant activity and metabolite levels of cucumber peel and flesh according to Pearson’s correlation coefficient. Each square indicates Pearson’s correlation coefficient values (*r*). Red and blue represent positive (0 < *r* < 1) and negative (-1 < *r* < 0) correlation, respectively. Asterisks indicated *p* < 0.05.


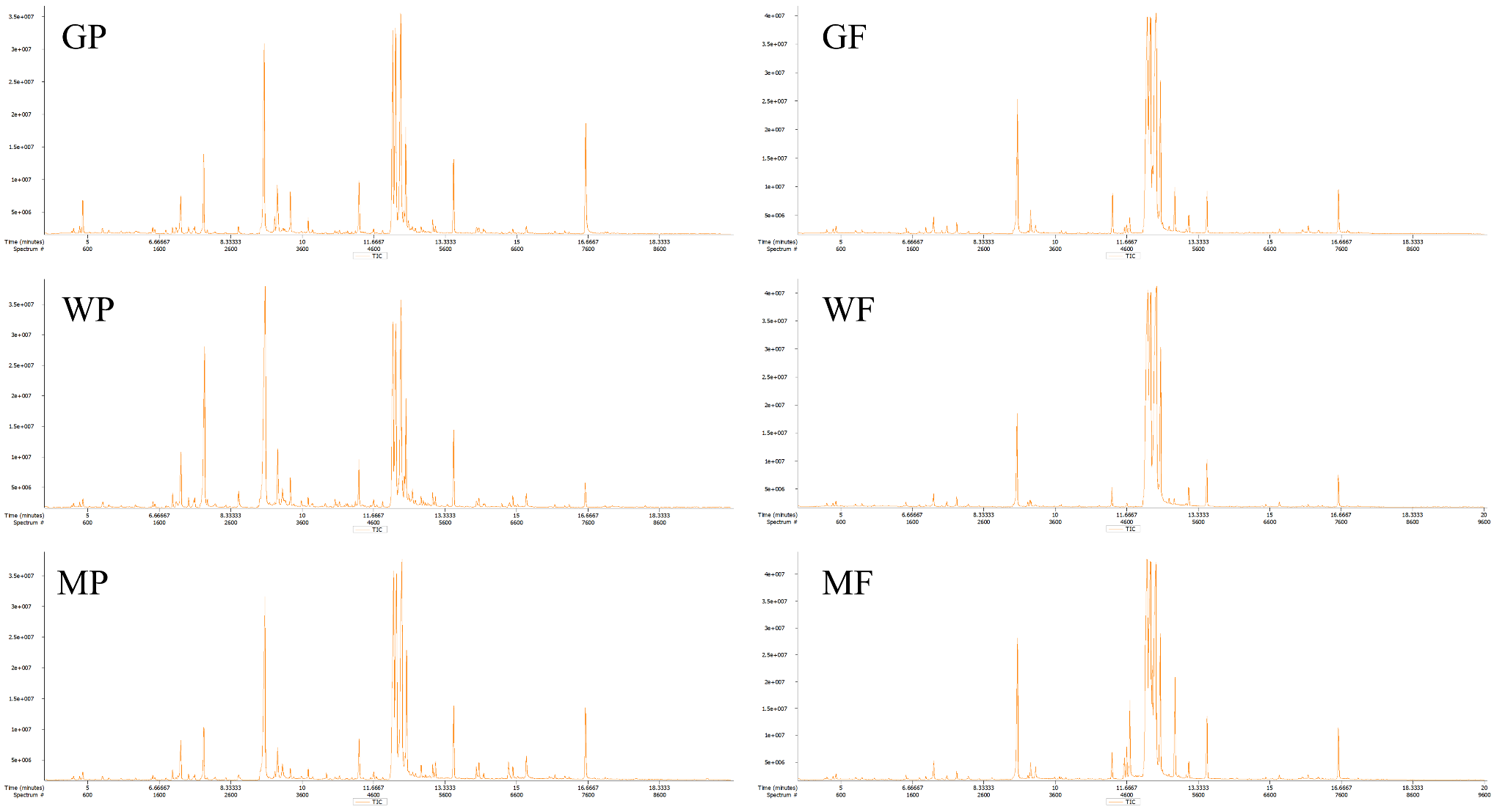


**Figure S4.** Chromatographs of GC-TOF-MS analysis. GP, Chuichung peel; WP, White Dadagi peel; MP, Mini peel; GF, Chuichung flesh; WF, White Dadagi flesh; MF, Mini flesh.


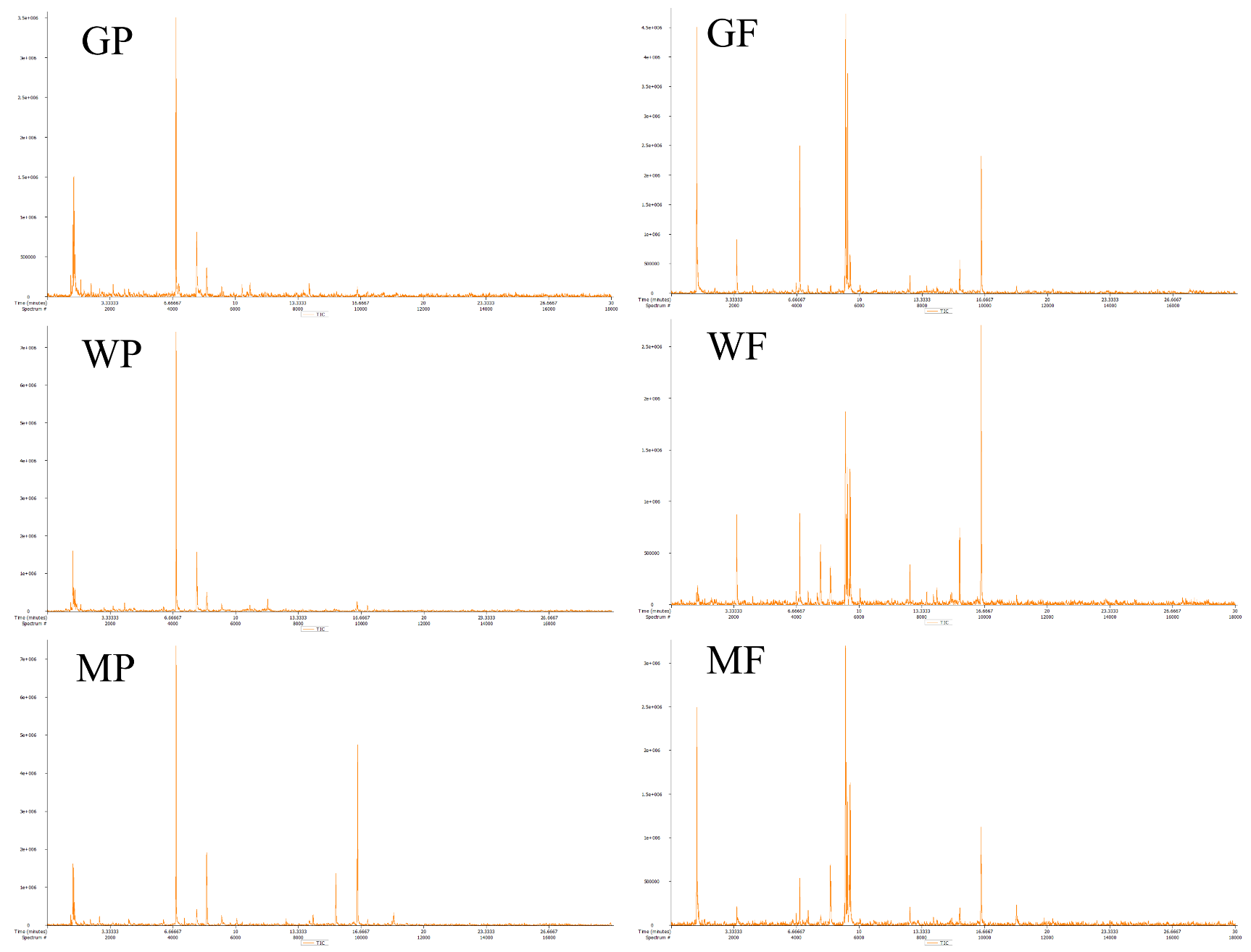


**Figure S5.** Chromatographs of SPME-GC-TOF-MS analysis. GP, Chuichung peel; WP, White Dadagi peel; MP, Mini peel; GF, Chuichung flesh; WF, White Dadagi flesh; MF, Mini flesh.


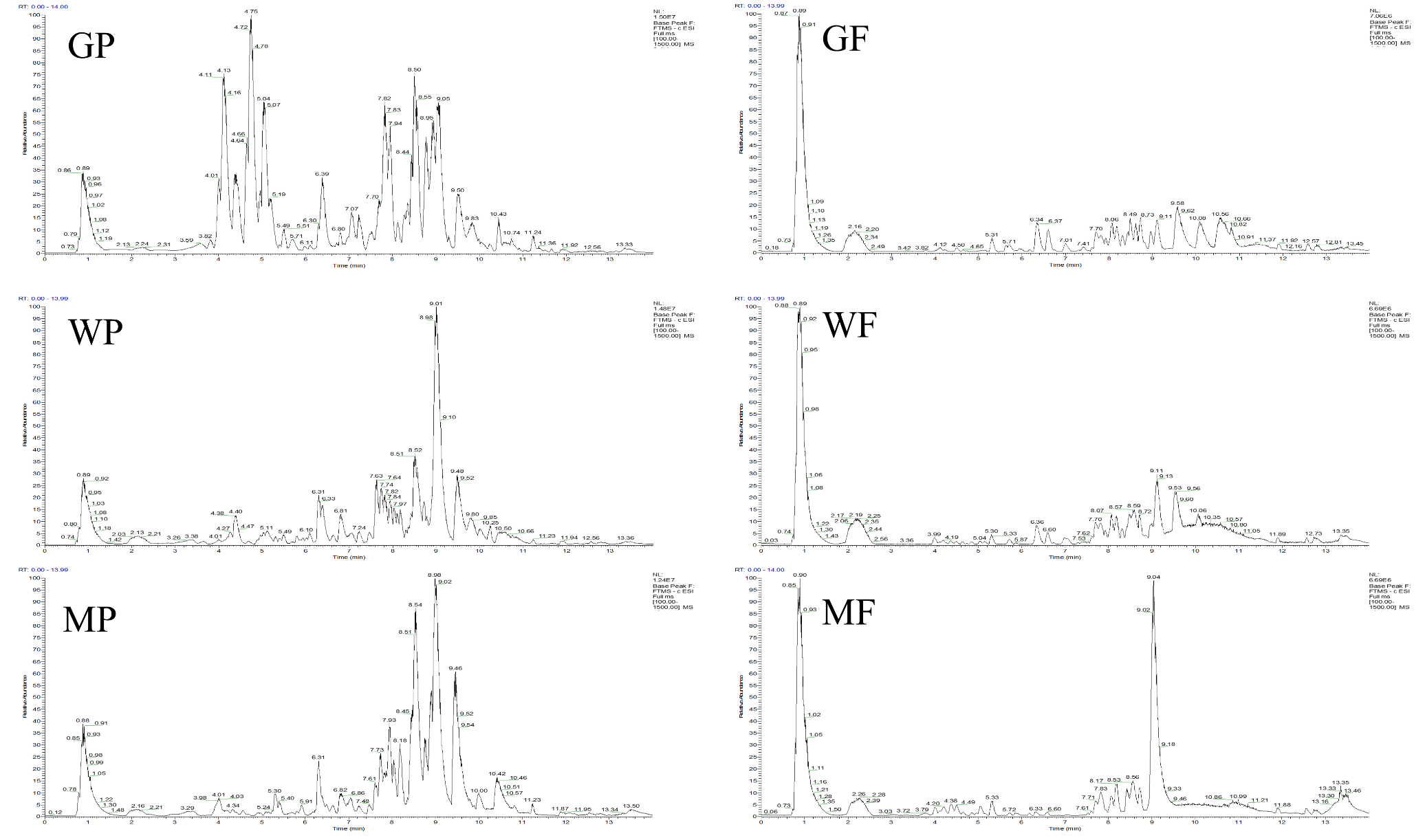


**Figure S6.** Chromatographs of UHPLC-LTQ-orbitrap-MS/MS analysis. GP, Chuichung peel; WP, White Dadagi peel; MP, Mini peel; GF, Chuichung flesh; WF, White Dadagi flesh; MF, Mini flesh.

**
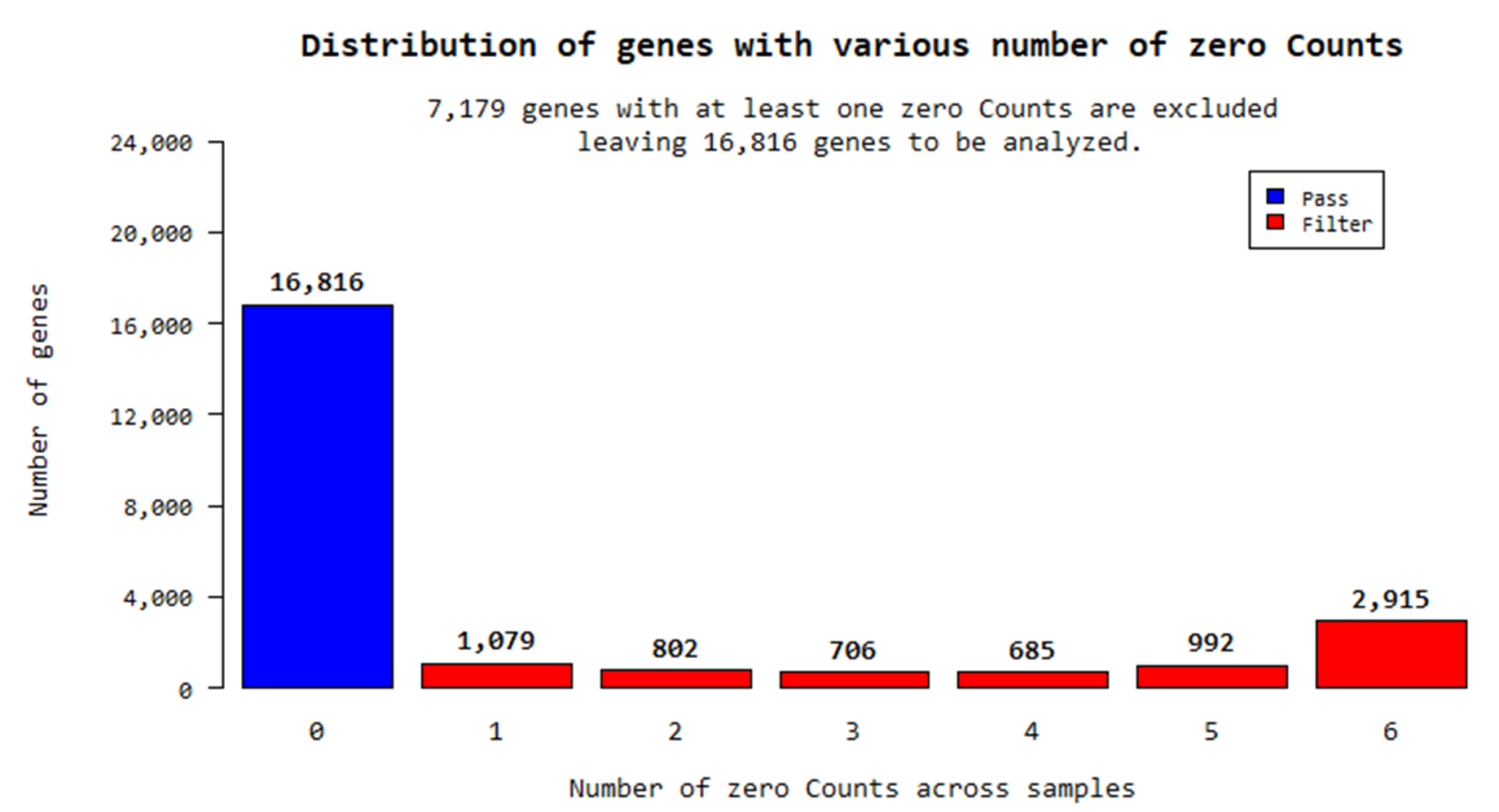
**

**Figure S7.** Distribution of genes with various number of zero counts.


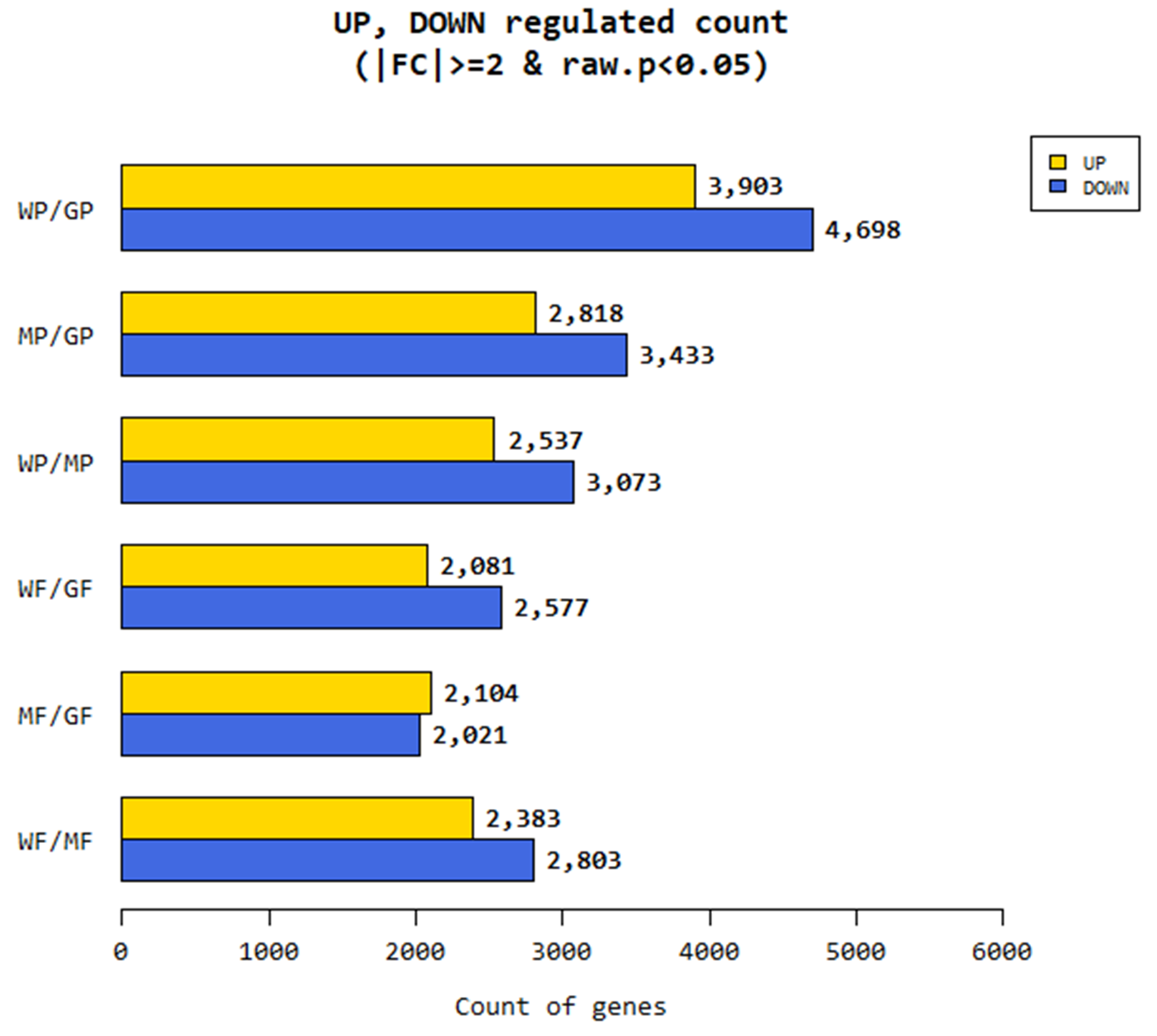


**Figure S8.** Count of genes regulated up and down in cucumber samples based on $\left| FC \right|$ ≥ 2 and *p* < 0.05. FC, fold change; GP, Chuichung peel; WP, White Dadagi peel; MP, Mini peel; GF, Chuichung flesh; WF, White Dadagi flesh; MF, Mini flesh.

**Table S1. Differential volatile organic compounds identified by HS-SPME-GC-TOF-MS in the peel of three different cucumbers.**

| **No.** | **Tentative Identification^a^** | **HS-SPME-GC-TOF-MS** | | | | | |  |
| --- | --- | --- | --- | --- | --- | --- | --- | --- |
|  |  | **Ret (min)^b^** | **Unique mass(*m/z*)** | **MS fragment pattern(*m/z*)** | **CAS** | **Molecular Formula** | **Weight** | **ID** |
| *Aldehydes* | | | | | | | | |
| 1 | Propanal | 1.41 | 58 | 58, 57, 55, 56, 59, 53, 45, 54 | 123-38-6 | C3H6O | 58 | Lib/MS^C^ |
| 2 | *(E)*-2-Pentenal | 2.92 | 83 | 55, 83, 84, 53, 56, 50, 51, 69 | 1576-87-0 | C5H8O | 84 | Lib/MS |
| 3 | Hexanal | 3.48 | 56 | 56, 57, 45, 55, 72, 67, 58, 82 | 66-25-1 | C6H12O | 100 | Lib/MS |
| 4 | 2-Hexenal | 4.32 | 98 | 55, 69, 57, 83, 56, 70, 53, 54 | 6728-26-3 | C6H10O | 98 | Lib/MS |
| 5 | Heptanal | 5.11 | 70 | 55, 70, 57, 45, 81, 71, 68, 67 | 111-71-7 | C7H14O | 114 | Lib/MS |
| 6 | 2,4-Heptadienal | 6.76 | 81 | 81, 53, 67, 55, 68, 79, 51, 110 | 881395 | C7H10O | 110 | Lib/MS |
| 7 | *(E)*-2-Nonenal | 7.29 | 69 | 69, 55, 57, 70, 56, 68, 83, 81 | 18829-56-6 | C9H16O | 140 | Lib/MS |
| 8 | *(E)*-6-Nonenal | 8.44 | 98 | 55, 67, 54, 81, 71, 79, 70, 69 | 2277-20-5 | C9H16O | 140 | Lib/MS |
| 9 | *(Z)*-6-Nonenal | 8.48 | 93 | 55, 54, 67, 81, 70, 57, 79, 69 | 2277-19-2 | C9H16O | 140 | Lib/MS |
| 10 | *(E,Z)*-2,6-Nonadienal | 9.12 | 109 | 70, 69, 67, 79, 81, 68, 53, 95 | 557-48-2 | C9H14O | 138 | Lib/MS |
| 11 | Nonenal | 9.37 | 70 | 55, 70, 57, 69, 83, 56, 67, 81 | 2277-19-2 | C9H16O | 140 | Lib/MS |
| 12 | Decanal | 10.04 | 57 | 57, 55, 56, 67, 68, 70, 71, 82 | 112-31-2 | C10H20O | 156 | Lib/MS |
| 13 | Tridecanal | 14.14 | 154 | 57, 55, 82, 67, 68, 69, 56, 81 | 10486-19-8 | C13H26O | 198 | Lib/MS |
| 14 | Tetradecanal | 15.34 | 168 | 57, 55, 82, 67, 68, 69, 56, 81 | 124-25-4 | C14H28O | 212 | Lib/MS |
| *Alcohols* | | | | | | | | |
| 15 | 1-Heptanol | 6.29 | 70 | 55, 56, 77, 106, 105, 69, 57, 51 | 111-70-6 | C7H16O | 116 | Lib/MS |
| *Sesquiterpenoids* | | | | | | | | |
| 16 | α-Humulene | 13.60 | 93 | 93, 80, 91, 79, 67, 92, 77, 121 | 6753-98-6 | C15H24 | 204 | Lib/MS |
| 17 | β-Ionone | 13.94 | 177 | 57, 71, 123, 85, 55, 56, 177, 69 | 79-77-6 | C13H20O | 192 | Lib/MS |
| *Ketones* | | | | | | | | |
| 18 | 3,5-Octadien-2-one | 7.99 | 95 | 95, 55, 56, 81, 79, 53, 70, 69 | 38284-27-4 | C8H12O | 124 | Lib/MS |
| *Furans* | | | | | | | | |
| 19 | 2-Pentyl-furan | 6.65 | 81 | 81, 82,53, 138, 51, 95, 94, 68 | 3777-69-3 | C9H14O | 138 | Lib/MS |
| *Alkanes* | | | | | | | | |
| 20 | Tetradecane | 12.69 | 57 | 57, 71, 85, 55, 56, 70, 69, 99 | 629-59-4 | C14H30 | 198 | Lib/MS |
| *Non-identification* | | | | | | | | |
| 21 | N.I.^d^ (1) | 3.48 | 67 | 67, 55, 52, 53, 77, 69, 79 |  |  |  |  |
| 22 | N.I. (2) | 5.44 | 122 | 107, 122, 108, 65, 51, 71, 340 |  |  |  |  |
| 23 | N.I. (3) | 8.36 | 137 | 52, 137, 124, 63, 109, 75, 108 |  |  |  |  |
| 24 | N.I. (4) | 8.58 | 267 | 267, 53, 79, 67, 126, 68, 65 |  |  |  |  |

^a^Identified volatile organic compounds based on the VIP value (> 0.7) and *p* < 0.05 from the PLS-DA model in Figure 3C; ^b^Retention time; ^c^MS, mass spectrum compared with the National Institute of Standards and Technology (NIST) database and in-house libraries; ^d^N.I., Non-identified

**Table S2. Differential primary metabolites identified by GC-TOF-MS in the peel of three different cucumbers.**

| **No.** | **Tentative Identification^a^** | **GC-TOF-MS** | | | | |
| --- | --- | --- | --- | --- | --- | --- |
|  |  | **Ret (min)^b^** | **Unique mass(*m/z*)** | **MS fragment pattern(*m/z*)** | **TMS** | **ID** |
| *Amino acids & amines* | | | | | | |
| 1 | Alanine | 5.34 | 116 | 73,116,147,59,75,117,77,74,103,100,58 | 2 | Lib/STD |
| 2 | Valine | 6.57 | 144 | 73,144,147,59,218,75,100,74,145,72,103 | 2 | Lib/STD |
| 3 | Ethanolamine | 7.07 | 174 | 73,174,86,100,59,147,175,75,74,133,130 | 3 | Lib/STD |
| 4 | Leucine | 7.12 | 158 | 73,158,59,100,102,74,159,57,86,160,130 | 2 | Lib/STD |
| 5 | Isoleucine | 7.34 | 158 | 73,158,74,218,100,59,159,147,58,86,103 | 2 | Lib/STD |
| 6 | Aspartic acid | 9.37 | 232 | 73,232,100,75,147,74,218,117,59,233,133 | 3 | Lib/STD |
| 7 | Proline | 9.42 | 156 | 73,156,75,147,157,74,59,58,84,258,230 | 2 | Lib/STD |
| 8 | GABA | 9.44 | 174 | 73,174,147,75,86,59,100,175,74,304,133 | 3 | Lib/STD |
| 9 | Glutamic acid | 10.14 | 246 | 73,75,246,84,128,147,56,156,74,59,247 | 3 | Lib/STD |
| 10 | Phenylalanine | 10.24 | 218 | 73,218,192,100,75,147,74,91,219,59,120 | 2 | Lib/STD |
| 11 | Glutamine | 11.34 | 156 | 73,75,156,74,147,157,59,77,131,56,155 | 3 | Lib/STD |
| 12 | Lysine | 12.37 | 174 | 174,156,128,76,230,175,317,176,200,154,318 | 4 | Lib/STD |
| 13 | Tyrosine | 12.49 | 179 | 73,218,219,100,179,147,74,220,280,77,180 | 3 | Lib/MS |
| 14 | Tryptophan | 14.26 | 202 | 73,202,203,74,291,204,59,130,147,145,103 | 3 | Lib/STD |
| *Organic acids* | | | | | | |
| 15 | Lactic acid | 4.89 | 117 | 73,147,117,66,75,77,59,74,148,88,190 | 2 | Lib/STD |
| 16 | Malonic acid | 6.44 | 147 | 147,73,75,66,148,77,72,74,59,143,133 | 2 | Lib/STD |
| 17 | Succinic acid | 7.50 | 247 | 147,73,75,55,56,148,77,247,149,129,74 | 2 | Lib/STD |
| 18 | Fumaric acid | 7.79 | 245 | 73,245,75,147,143,246,53,115,133,83,59 | 2 | Lib/STD |
| 19 | Malic acid | 9.12 | 133 | 73,147,55,75,74,133,59,101,148,117,149 | 3 | Lib/STD |
| 20 | 2-Hydroxyglutaric acid | 9.79 | 247 | 129,77,75,247,155,85,156,203,133,56,78 | 3 | Lib/MS |
| 21 | Citric acid | 11.67 | 273 | 73,147,273,75,67,133,74,148,274,211,72 | 4 | Lib/STD |
| *Sugars & Sugar alcohols* | | | | | | |
| 22 | Glycerol | 7.15 | 218 | 73,147,117,103,205,133,59,75,74,148,101 | 3 | Lib/STD |
| 23 | Glyceric acid | 7.71 | 189 | 73,147,103,133,75,189,102,59,117,74,101 | 3 | Lib/MS |
| 24 | Threonic acid | 9.72 | 292 | 73,147,117,75,103,292,74,220,55,102,205 | 4 | Lib/STD |
| 25 | Xylose | 10.51 | 103 | 103,217,147,73,75,133,117,307,104,189,160 | 4 | Lib/STD |
| 26 | Xylitol | 10.98 | 217 | 73,147,103,217,117,129,205,75,74,59,133 | 5 | Lib/STD |
| 27 | Ribitol | 11.02 | 217 | 73,217,103,129, 205,133,218,189,101 | 5 | Lib/STD |
| 28 | Fructose | 12.14 | 103 | 73,103,147,74,117,133,89,217,59,148 | 5 | Lib/STD |
| 29 | Galactose | 12.34 | 160 | 73,160,147,74,103,205,75,320,148,161,217 | 5 | Lib/STD |
| 30 | Glucose | 12.44 | 160 | 73,103,147,160,74,117,133,89,59,75,129 | 5 | Lib/STD |
| 31 | myo-inositol | 13.54 | 191 | 73,147,217,191,74,133,129,103,75,147,204 | 6 | Lib/STD |
| *Fatty acids* | | | | | | |
| 32 | Palmitic acid | 13.05 | 117 | 73,117,75,132,129,55,145,118,131,313,61 | 1 | Lib/STD |
| 33 | Linoleic acid | 14.07 | 81 | 75,73,67,55,81,129,77,117,95,79,54 | 1 | Lib/STD |
| 34 | Oleamide | 15.23 | 144 | 75,73,131,116,128,144,55,77,74,91,69 | 1 | Lib/STD |

^a^Identified metabolites based on the VIP value (> 0.7) and *p* < 0.05 from the PLS-DA model in Figure 3A; ^b^Retention time; TMS, Trimethylsilyl; MS, mass spectrum compared with the National Institute of Standards and Technology (NIST) database and in-house libraries; STD, mass spectrum consistent with that of the standard compounds.

**Table S3. Tentative identified secondary metabolites in the peel of three different cucumbers based on UHPLC-LTQ-Orbitrap-MS/MS**

| **No.** | **Tentative Identification^a^** | **UHPLC-LTQ-Orbitrap-MS/MS** | | | | | | |  |
| --- | --- | --- | --- | --- | --- | --- | --- | --- | --- |
|  |  | **Ret (min)^b^** | **[M‒H]^‒^** | **[M+H]^+^** | **M.W.** | **Molecular  Formula** | **Error (ppm)** | **MS^n^ fragment pattern (*m/z*)^c^** | **ID** |
| *Flavonoids* | | | | | | | | | |
| 1 | Saponarin 4'-*O*-glucoside | 4.01 | 755.2035 | 757.2172 | 756 | C33H40O20 | -0.684 | (‒)755>593, 455, 413 | Kitajima et al. 2003 |
| 2 | Quercetin 7-*O*-glucoside-3-*O*-rutinoside | 4.20 | 771.1987 | 773.2138 | 772 | C33H40O21 | -0.261 | (‒)771>609 | Brito et al. 2014 |
| 3 | Vicenin-2 | 4.66 | 593.1501 | 595.1645 | 594 | C27H30O15 | -1.843 | (‒)593>575, 413> 353 | Santos et al. 2012 |
| 4 | Lucenin-2-methyl ether | 4.74 | 623.1597 | 625.1743 | 624 | C28H32O16 | -3.302 | (‒)623>605, 503>413, 383 | Abu Reidah, 2013 |
| 5 | Apigetrin | 4.87 | 431.0981 | 433.1119 | 432 | C21H20O10 | -0.626 | (‒)431>341, 311, 269 | Ul Haq et al. 2019 |
| 6 | Scoparin | 4.96 | 461.1082 | 463.1224 | 462 | C22H22O11 | -1.702 | (‒)461>341>298 | Brito et al. 2014 |
| 7 | Isovitexin 2''-*O*- (6'''-feruloyl)glucoside | 5.05 | 769.1960 | 771.2118 | 770 | C37H38O18 | -3.299 | (‒)769>751>323, 308 | Zhao et al. 2016 |
| 8 | Isovitexin 2''-*O*- (6'''-(*E*)-*p*-coumaroyl)glucoside | 5.07 | 739.1869 | 741.2013 | 740 | C36H36O17 | -1.451 | (‒)739>707,575>293 | Zhao et al. 2016 |
| 9 | Isorhamnetin-3-*O*-glucoside | 5.24 | 477.1038 | 479.1174 | 478 | C22H22O12 | -0.103 | (‒)477>314>300, 270 | Du et al. 2014 |
| 10 | Homoeriodictyol | 6.50 | 301.0720 | 303.0855 | 302 | C16H14O6 | 0.660 | (‒)301>286>134 | Lévèques et al. 2012 |
| *Hydroxycinnamate derivatives* | |  |  |  |  |  |  |  |  |
| 11 | *p*-Coumaric acid-*O*-glucoside | 3.67 | 325.0928 | ‒ | 326 | C15H18O8 | -0.279 | (‒)325>163>119 | Moco et al. 2006 |
| 12 | Feruloyl glucose | 3.71 | 355.1039 | ‒ | 356 | C16H20O9 | 1.252 | (‒)355>311, 193 | Serafini et al. 2002 |
| 13 | Sinapic acid-hexoside | 3.97 | 385.1136 | ‒ | 386 | C17H22O10 | -1.091 | (‒)385>367, 223>208 | Abu-Reidah et al. 2012 |
| *Lipids* |  |  |  |  |  |  |  |  |  |
| 14 | LysoPE(18:3) | 8.04 | 474.2615 | 476.2758 | 475 | C23H42NO7P | -2.345 | (‒)474>456 | Suárez-García et al. 2017 |
| 15 | LysoPC(18:3) | 8.19 | 562.3135^e^ | 518.3225 | 517 | C26H48NO7P | -3.117 | (+)518>184 | Suárez-García et al. 2017 |
| 16 | LysoPE(18:2) | 8.44 | 476.2776 | 478.2918 | 477 | C23H44O7NP | -2.123 | (+)478>460, 337 | Suárez-García et al. 2017 |
| 17 | LysoPI(18:1) | 8.53 | 597.3034 | - | 598 | C27H51O12P | -1.886 | (‒)597>417,315,281 | Chen et al. 2017 |
| 18 | LysoPE(16:0) | 8.72 | 452.2775 | 454.2917 | 453 | C21H44NO7P | -2.456 | (+)454>436, 313 | Suárez-García et al. 2017 |
| 19 | LysoPC(16:0) | 8.97 | 540.3295^e^ | 496.3386 | 495 | C24H50NO7P | -2.349 | (+)496>184 | Suárez-García et al. 2017 |
| 20 | LysoPA(18:1) | 9.83 | 435.2530 | - | 436 | C21H41O7P | 2.933 | (‒)435>153 | Suárez-García et al. 2017 |
| 21 | 9-OxoOTrE | 8.76 | 291.1966 | 293.2104 | 292 | C18H28O3 | 0.110 | (‒)291>273>185 | Zhao et al. 2016 |
| *Non-identification* | |  |  |  |  |  |  |  |  |
| 22 | N.I.^d^ 1 | 4.12 | 785.2118 | 787.2273 | 786 | ‒ | ‒ | (‒)785>769> 607 | ‒ |
| 23 | N.I. 2 | 6.04 | 226.1085 | ‒ | 227 | ‒ | ‒ | (‒)226>182>164 | ‒ |
| 24 | N.I. 3 | 7.07 | 341.2325 | 365.2290^f^ | 342 | ‒ | ‒ | (‒)341>323>273 | ‒ |
| 25 | N.I. 4 | 7.64 | 235.1704 | 237.1843 | 236 | ‒ | ‒ | (‒)235>217 | ‒ |
| 26 | N.I. 5 | 7.64 | 253.1806 | 255.1949 | 254 | ‒ | ‒ | (‒)253>235>173 | ‒ |
| 27 | N.I. 6 | 7.81 | 675.3578 | 699.3543^f^ | 676 | ‒ | ‒ | (‒)675>657>217 | ‒ |
| 28 | N.I. 7 | 7.82 | 291.1966 | 293.2104 | 292 | ‒ | ‒ | (‒)291>273>255,213 | ‒ |
| 29 | N.I. 8 | 8.44 | 614.3291 | 616.3441 | 615 | ‒ | ‒ | (‒)614>596>434,237 | ‒ |
| 30 | N.I. 9 | 8.44 | 559.3111^e^ | 537.3019^f^ | 514 | ‒ | ‒ | (‒)559>541 | ‒ |
| 31 | N.I. 10 | 10.27 | 295.2278^e^ | 251.2364 | 250 | ‒ | ‒ | (‒)295>277>137 | ‒ |
| 32 | N.I. 11 | 10.44 | 997.5703^e^ | 975.5623^f^ | 952 | ‒ | ‒ | (‒)997>978>933,305 | ‒ |
| 33 | N.I. 12 | 10.62 | 995.5562^e^ | 973.5462^f^ | 950 | ‒ | ‒ | (‒)995>977,949>829,361 | ‒ |

^a^Identified metabolites based on the VIP value (>0.7) and *p* <0.05 from the PLS-DA model in Figure 3B; ^b^Retention time; ^c^MS^n^ fragment patterns detected in negative or positive ion mode.; ^d^N.I., Non-identified; ^e^[M+FA‒H]^‒^; ^f^[M+Na]^+^ -, not detected.

**Table S4. Differential volatile organic compounds identified by HS-SPME-GC-TOF-MS in the flesh of three different cucumbers.**

| **No.** | **Tentative Identification^a^** | **HS-SPME-GC-TOF-MS** | | | | | | |
| --- | --- | --- | --- | --- | --- | --- | --- | --- |
|  |  | **Ret (min)^b^** | **Unique mass(m/z)** | **MS fragment pattern(m/z)** | **CAS** | **Molecular Formula** | **Weight** | **ID** |
| *Aldehydes* | | | | | | | | |
| 1 | Propanal | 1.41 | 58 | 58, 57, 55, 56, 59, 53, 45, 54 | 123-38-6 | C3H6O | 58 | Lib/MS^C^ |
| 2 | *(E)*-2-Pentenal | 2.92 | 83 | 55, 83, 84, 53, 56, 50, 51, 69 | 1576-87-0 | C5H8O | 84 | Lib/MS |
| 3 | Hexanal | 3.48 | 56 | 56, 57, 45, 55, 72, 67, 58, 82 | 66-25-1 | C6H12O | 100 | Lib/MS |
| 4 | 2-Hexenal | 4.32 | 98 | 55, 69, 57, 83, 56, 70, 53, 54 | 6728-26-3 | C6H10O | 98 | Lib/MS |
| 5 | Heptanal | 5.11 | 70 | 55, 70, 57, 45, 81, 71, 68, 67 | 111-71-7 | C7H14O | 114 | Lib/MS |
| 6 | 2-Heptenal | 6.06 | 83 | 55, 57, 83, 56, 69, 70, 68, 53 | 18829-55-5 | C7H12O | 112 | Lib/MS |
| 7 | 2,4-Heptadienal | 6.76 | 81 | 81, 53, 67, 55, 68, 79, 51,110 | 881395 | C7H10O | 110 | Lib/MS |
| 8 | *(E)*-2-Nonenal | 7.29 | 69 | 69, 55, 57, 70, 56, 68, 83, 81 | 18829-56-6 | C9H16O | 140 | Lib/MS |
| 9 | *(E)*-2-Octenal | 7.76 | 70 | 55, 70, 57, 83, 69, 67, 82,58 | 2548-87-0 | C8H14O | 126 | Lib/MS |
| 10 | *(E)*-6-Nonenal | 8.44 | 98 | 55, 67, 54, 81, 71, 79, 70, 69 | 2277-20-5 | C9H16O | 140 | Lib/MS |
| 11 | *(Z)*-6-Nonenal | 8.48 | 93 | 55, 54, 67, 81, 70, 57, 79, 69 | 2277-19-2 | C9H16O | 140 | Lib/MS |
| 12 | *(E,Z)*-2,6-Nonadienal | 9.12 | 109 | 70, 69, 67, 79, 81, 68, 53, 95 | 557-48-2 | C9H14O | 138 | Lib/MS |
| 13 | Nonenal | 9.37 | 70 | 55, 70, 57, 69, 83, 56, 67, 81 | 2277-19-2 | C9H16O | 140 | Lib/MS |
| 14 | Decanal | 10.04 | 57 | 57, 55, 56, 67, 68, 70, 71, 82 | 112-31-2 | C10H20O | 156 | Lib/MS |
| 15 | Tridecanal | 14.14 | 154 | 57, 55, 82, 67, 68, 69, 56, 81 | 10486-19-8 | C13H26O | 198 | Lib/MS |
| 16 | Tetradecanal | 15.34 | 168 | 57, 55, 82, 67, 68, 69, 56, 81 | 124-25-4 | C14H28O | 212 | Lib/MS |
| *Alcohols* | | | | | | | | |
| 17 | 1-Heptanol | 6.29 | 70 | 55, 56, 77, 106, 105, 69, 57, 51 | 111-70-6 | C7H16O | 116 | Lib/MS |
| 18 | 1-Nonanol | 9.52 | 56 | 55, 56, 67, 70, 69, 68, 57, 82 | 143-08-8 | C9H20O | 144 | Lib/MS |
| 19 | β-Bisabolene | 14.18 | 93 | 69, 93, 67, 57, 55, 68, 82, 91 | 25532-79-0 | C15H24 | 204 | Lib/MS |
| *Sesquiterpenoids* | | | | | | | | |
| 20 | α-Humulene | 13.60 | 93 | 93, 80, 91, 79, 67, 92, 77, 121 | 6753-98-6 | C15H24 | 204 | Lib/MS |
| 21 | β-Ionone | 13.94 | 177 | 57, 71, 123, 85, 55, 56, 177, 69 | 79-77-6 | C13H20O | 192 | Lib/MS |
| *Ketones* | | | | | | | | |
| 22 | 3,5-Octadien-2-one | 7.99 | 95 | 95, 55, 56, 81, 79, 53, 70, 69 | 38284-27-4 | C8H12O | 124 | Lib/MS |
| *Furans* | | | | | | | | |
| 23 | 2-Pentyl-furan | 6.65 | 81 | 81, 82,53, 138, 51, 95, 94, 68 | 3777-69-3 | C9H14O | 138 | Lib/MS |
| *Alkanes* | | | | | | | | |
| 24 | Tetradecane | 12.69 | 57 | 57, 71, 85, 55, 56, 70, 69, 99 | 629-59-4 | C14H30 | 198 | Lib/MS |
| 25 | Pentadecane | 13.95 | 113 | 57, 71, 55, 85, 56, 177, 70, 69 | 629-62-9 | C15H32 | 212 | Lib/MS |
| *Non-identification* | | | | | | | | |
| 26 | N.I.^d^ (1) | 3.48 | 67 | 67, 55, 52, 53, 77, 69, 79 |  |  |  |  |
| 27 | N.I. (2) | 5.44 | 122 | 107, 122, 108, 65, 51, 71, 340 |  |  |  |  |
| 28 | N.I. (3) | 8.36 | 137 | 52, 137, 124, 63, 109, 75, 108 |  |  |  |  |
| 29 | N.I. (4) | 8.58 | 267 | 267, 53, 79, 67, 126, 68, 65 |  |  |  |  |

^a^Identified volatile organic compounds based on the VIP value (> 0.7) and *p* < 0.05 from the PLS-DA model in Figure 4C; ^b^Retention time; ^c^MS, mass spectrum compared with the National Institute of Standards and Technology (NIST) database and in-house libraries; ^d^N.I., Non-identified

**Table S5. Differential primary metabolites identified by GC-TOF-MS in the flesh of three different cucumbers.**

| **No.** | **Tentative Identification^a^** | **GC-TOF-MS** | | | | |
| --- | --- | --- | --- | --- | --- | --- |
|  |  | **Ret (min)^b^** | **Unique mass(m/z)** | **MS fragment pattern(m/z)** | **TMS** | **ID** |
| *Amino acids & amines* | | | | | | |
| 1 | Valine | 6.57 | 144 | 73,144,147,59,218,75,100,74,145,72,103 | 2 | Lib/STD |
| 2 | Ethanolamine | 7.07 | 174 | 73,174,86,100,59,147,175,75,74,133,130 | 3 | Lib/STD |
| 3 | Leucine | 7.12 | 158 | 73,158,59,100,102,74,159,57,86,160,130 | 2 | Lib/STD |
| 4 | Isoleucine | 7.34 | 158 | 73,158,74,218,100,59,159,147,58,86,103 | 2 | Lib/STD |
| 5 | Glycine | 7.47 | 174 | 73,174,86,147,59,100,175,133,248,74,75 | 3 | Lib/STD |
| 6 | Serine | 7.97 | 218 | 73,204,218,100,75,147,59,116,74,205,77 | 3 | Lib/STD |
| 7 | Aspartic acid | 9.37 | 232 | 73,232,100,75,147,74,218,117,59,233,133 | 3 | Lib/STD |
| 8 | GABA | 9.44 | 174 | 73,174,147,75,86,59,100,175,74,304,133 | 3 | Lib/STD |
| 9 | Glutamic acid | 10.14 | 246 | 73,75,246,84,128,147,56,156,74,59,247 | 3 | Lib/STD |
| 10 | Phenylalanine | 10.24 | 218 | 73,218,192,100,75,147,74,91,219,59,120 | 2 | Lib/STD |
| 11 | Lysine | 12.37 | 174 | 174,156,128,76,230,175,317,176,200,154,318 | 4 | Lib/STD |
| 12 | Tryptophan | 14.26 | 202 | 73,202,203,74,291,204,59,130,147,145,103 | 3 | Lib/STD |
| *Organic acids* | | | | | | |
| 13 | Lactic acid | 4.89 | 117 | 73,147,117,66,75,77,59,74,148,88,190 | 2 | Lib/STD |
| 14 | Malonic acid | 6.44 | 147 | 147,73,75,66,148,77,72,74,59,143,133 | 2 | Lib/STD |
| 15 | Succinic acid | 7.50 | 247 | 147,73,75,55,56,148,77,247,149,129,74 | 2 | Lib/STD |
| 16 | Fumaric acid | 7.79 | 245 | 73,245,75,147,143,246,53,115,133,83,59 | 2 | Lib/STD |
| 17 | Malic acid | 9.12 | 133 | 73,147,55,75,74,133,59,101,148,117,149 | 3 | Lib/STD |
| 18 | 2-Hydroxyglutaric acid | 9.79 | 247 | 129,77,75,247,155,85,156,203,133,56,78 | 3 | Lib/MS |
| 19 | Citric acid | 11.67 | 273 | 73,147,273,75,67,133,74,148,274,211,72 | 4 | Lib/STD |
| *Sugars & Sugar alcohols* | | | | | | |
| 20 | Glyceric acid | 7.71 | 189 | 73,147,103,133,75,189,102,59,117,74,101 | 3 | Lib/MS |
| 21 | Threonic acid | 9.72 | 292 | 73,147,117,75,103,292,74,220,55,102,205 | 4 | Lib/STD |
| 22 | Xylose | 10.51 | 103 | 103,217,147,73,75,133,117,307,104,189,160 | 4 | Lib/STD |
| 23 | Xylitol | 10.98 | 217 | 73,147,103,217,117,129,205,75,74,59,133 | 5 | Lib/STD |
| 24 | Ribitol | 11.02 | 217 | 73,217,103,129, 205,133,218,189,101,204,206,, | 5 | Lib/STD |
| 25 | Fructose | 12.14 | 103 | 73,103,147,74,117,133,89,217,59,148 | 5 | Lib/STD |
| 26 | Galactose | 12.34 | 160 | 73,160,147,74,103,205,75,320,148,161,217 | 5 | Lib/STD |
| 27 | Glucose | 12.44 | 160 | 73,103,147,160,74,117,133,89,59,75,129 | 5 | Lib/STD |
| 28 | myo-inositol | 13.54 | 191 | 73,147,217,191,74,133,129,103,75,147,204 | 6 | Lib/STD |
| *Fatty acids* | | | | | | |
| 29 | Linoleic acid | 14.07 | 81 | 75,73,67,55,81,129,77,117,95,79,54 | 1 | Lib/STD |

^a^Identified metabolites based on the VIP value (> 0.7) and *p* < 0.05 from the PLS-DA model in Figure 4A; ^b^Retention time; TMS, Trimethylsilyl; MS, mass spectrum compared with the National Institute of Standards and Technology (NIST) database and in-house libraries; STD, mass spectrum consistent with that of the standard compounds.

**Table S6. Tentative identified secondary metabolites in the flesh of three different cucumbers based on UHPLC-LTQ-Orbitrap-MS/MS**

| **No.** | **Tentative Identification^a^** | **UHPLC-LTQ-Orbitrap-MS/MS** | | | | | | |  |
| --- | --- | --- | --- | --- | --- | --- | --- | --- | --- |
|  |  | **Ret (min)^b^** | **[M‒H]^‒^** | **[M+H]^+^** | **M.W.** | **Molecular Formula** | **Error (ppm)** | **MS^n^ fragment pattern (m/z)^c^** | **ID** |
| *Flavonoids* | |  |  |  |  |  |  |  |  |
| 1 | Saponarin 4'-O-glucoside | 4.01 | 755.2035 | 757.2172 | 756 | C33H40O20 | -0.684 | (-)755>593, 455, 413 | Kitajima et al. 2003 |
| 2 | Quercetin 7-O-glucoside-3-O-rutinoside | 4.2 | 771.1987 | 773.2138 | 772 | C33H40O21 | -0.261 | (-)771>609 | Brito et al. 2014 |
| 3 | Eriodictyol-7-O-glucoside | 4.3 | 449.1100 | 451.1214 | 450 | C21H22O11 | 2.261 | (-)449>287 | Abu Reidah, 2013 |
| 4 | Vicenin-2 | 4.66 | 593.1501 | 595.1645 | 594 | C27H30O15 | -1.843 | (-)593>575, 413> 353 | Ul Haq et al. 2019 |
| 5 | Lucenin-2-methyl ether | 4.74 | 623.1597 | 625.1743 | 624 | C28H32O16 | -3.302 | (-)623>605, 503>413, 383 | Santos et al. 2012 |
| 6 | Apigetrin | 4.87 | 431.0981 | 433.1119 | 432 | C21H20O10 | -0.626 | (-)431>341, 311, 269 | Zhao et al. 2016 |
| 7 | Scoparin | 4.96 | 461.1082 | 463.1224 | 462 | C22H22O11 | -1.702 | (-)461>341>298 | Brito et al. 2014 |
| 8 | Isovitexin 2''-O-(6'''-feruloyl)glucoside | 5.05 | 769.1960 | 771.2118 | 770 | C37H38O18 | -3.299 | (-)769>751>323, 308 | Du et al. 2014 |
| 9 | Isovitexin 2''-O-(6'''-(E)-p-coumaroyl)glucoside | 5.07 | 739.1869 | 741.2013 | 740 | C36H36O17 | -1.451 | (-)739>707,575>293 | Du et al. 2014 |
| 10 | Isorhamnetin-3-O-glucoside | 5.24 | 477.1038 | 479.1174 | 478 | C22H22O12 | -0.103 | (-)477>314>300, 270 | Lévèques et al. 2012 |
| *Hydroxycinnamate derivatives* | |  |  |  |  |  |  |  |  |
| 11 | p-Coumaric acid-O-glucoside | 3.67 | 325.0928 | ‒ | 326 | C15H18O8 | -0.279 | (-)325>163>119 | Moco et al. 2006 |
| 12 | Feruloyl glucose | 3.71 | 355.1039 | ‒ | 356 | C16H20O9 | 1.252 | (-)355>311, 193 | Serafini et al. 2002 |
| 13 | Sinapic acid-hexoside | 3.97 | 385.1136 | ‒ | 386 | C17H22O10 | -1.091 | (-)385>367, 223>208 | Abu-Reidah et al. 2012 |
| *Lipids* | |  |  |  |  |  |  |  |  |
| 14 | 9-OxoOTrE | 8.76 | 291.1966 | 293.2104 | 292 | C18H28O3 | 0.11 | (-)291>273>185 | Zhao et al. 2016 |
| 15 | Lyso-PA(18:1) | 9.83 | 435.2530 | ‒ | 436 | C21H41O7P | 2.933 | (-)435>153 | Suárez-García et al. 2017 |
| *Non-identification* | |  |  |  |  |  |  |  |  |
| 16 | N.I.^d^ 1 | 4.12 | 785.2118 | 787.2273 | 786 | ‒ | ‒ | (-)785>769> 607 | ‒ |
| 17 | N.I. 3 | 7.07 | 341.2325 | 365.2290^f^ | 342 | ‒ | ‒ | (-)341>323>273 | ‒ |
| 18 | N.I. 5 | 7.64 | 253.1806 | 255.1949 | 254 | ‒ | ‒ | (-)253>235>173 | ‒ |
| 19 | N.I. 6 | 7.81 | 675.3578 | 699.3543^f^ | 676 | ‒ | ‒ | (-)675>657>217 | ‒ |
| 20 | N.I. 7 | 7.82 | 291.1966 | 293.2104 | 292 | ‒ | ‒ | (-)291>273>255,213 | ‒ |
| 21 | N.I. 13 | 8.06 | 638.3291 | 640.3439 | 639 | ‒ | ‒ | (-)559>476>433 | ‒ |
| 22 | N.I. 9 | 8.44 | 559.3111^e^ | 537.3019^f^ | 514 | ‒ | ‒ | (-)559>541 | ‒ |
| 23 | N.I. 11 | 10.44 | 997.5703^e^ | 975.5623^f^ | 952 | ‒ | ‒ | (-)997>978>933,305 | ‒ |
| 24 | N.I. 12 | 10.62 | 995.5562^e^ | 973.5462^f^ | 950 | ‒ | ‒ | (-)995>977,949>829,361 | ‒ |

^a^Identified metabolites based on the VIP value (> 0.7) and *p* < 0.05 from the PLS-DA model in Figure 4B; ^b^Retention time; ^c^MS^n^ fragment patterns detected in negative or positive ion mode.; ^d^N.I., Non-identified; ^e^[M+FA‒H]^‒^; ^f^ [M+Na]^+^; ‒, not detected.

**Table S7. Raw data statistics of RNA seq**

| **No.** | **Sample id** | **Raw data** | | | | |
| --- | --- | --- | --- | --- | --- | --- |
|  |  | **Total read bases** | **Total reads** | **GC(%)** | **Q20(%)** | **Q30(%)** |
| 1 | GF | 6,920,545,048 | 68,520,248 | 43.40 | 98.13 | 94.48 |
| 2 | GP | 6,197,631,084 | 61,362,684 | 43.58 | 98.04 | 94.31 |
| 3 | MF | 6,709,835,212 | 66,434,012 | 43.73 | 98.12 | 94.51 |
| 4 | MP | 7,001,427,060 | 69,321,060 | 43.05 | 98.66 | 95.74 |
| 5 | WF | 8,658,144,806 | 85,724,206 | 43.73 | 98.11 | 94.49 |
| 6 | WP | 6,298,733,296 | 62,363,696 | 43.15 | 98.54 | 95.54 |

^a^Number of total sequencing read bases; ^b^Number of total reads; ^c^contents of GC; ^d^percentage of bases with a phred quality score > 20; ^e^percentage of bases with a phred quality score > 30; GP, Chuichung peel; WP, White Dadagi peel; MP, Mini peel; GF, Chuichung flesh; WF, White Dadagi flesh; MF, Mini flesh.

**Table S8. Trimmed data statistics of RNA seq**

| **No.** | **Sample id** | **Trimmed data** | | | | |
| --- | --- | --- | --- | --- | --- | --- |
|  |  | **Total read bases** | **Total reads** | **GC(%)** | **Q20(%)** | **Q30(%)** |
| 1 | GF | 6,777,231,541 | 67,475,368 | 43.43 | 98.61 | 95.18 |
| 2 | GP | 6,062,934,751 | 60,348,194 | 43.61 | 98.56 | 95.06 |
| 3 | MF | 6,569,813,784 | 65,408,436 | 43.76 | 98.61 | 95.22 |
| 4 | MP | 6,901,709,325 | 68,629,182 | 43.07 | 99.00 | 96.24 |
| 5 | WF | 8,473,790,608 | 84,341,196 | 43.76 | 98.62 | 95.23 |
| 6 | WP | 6,203,252,666 | 61,682,094 | 43.18 | 98.92 | 96.08 |

^a^Number of total sequencing read bases after trimming; ^b^Number of total reads after trimming; ^c^contents of GC; ^d^percentage of bases with a phred quality score > 20; ^e^percentage of bases with a phred quality score > 30; GP, Chuichung peel; WP, White Dadagi peel; MP, Mini peel; GF, Chuichung flesh; WF, White Dadagi flesh; MF, Mini flesh.

**Table S9. Mapped data statistics**

| **No.** | **Sample id** | **# of processed reads** | **# of mapped reads (%)** | **# of unmapped reads (%)** |
| --- | --- | --- | --- | --- |
| 1 | GF | 67,475,368 | 66,897,706 (99.14%) | 577,662 (0.86%) |
| 2 | GP | 60,348,194 | 59,813,412 (99.11%) | 534,782 (0.89%) |
| 3 | MF | 65,408,436 | 59,294,839 (90.65%) | 6,113,597 (9.35%) |
| 4 | MP | 68,629,182 | 67,877,311 (98.90%) | 751,871 (1.10%) |
| 5 | WF | 84,341,196 | 83,584,104 (99.10%) | 757,092 (0.90%) |
| 6 | WP | 61,682,094 | 61,058,652 (98.99%) | 623,442 (1.01%) |

^a^Processed reads are indicated number of cleaned reads after trimming; ^b^Number of reads mapped to the reference; ^c^Number of reads unmapped to the reference. GP, Chuichung peel; WP, White Dadagi peel; MP, Mini peel; GF, Chuichung flesh; WF, White Dadagi flesh; MF, Mini flesh.

**Table S10. Differentially expressed genes (DEGs) related to carotenoid, chlorophyll, and flavonoid metabolism in three different cucumbers.**

| **No.** | **Gene ID** | **Annotation^a^** | **EC number^b^** | **Peel** | |  | **Flesh** | |
| --- | --- | --- | --- | --- | --- | --- | --- | --- |
|  |  |  |  | **C** | **M** |  | **C** | **M** |
| ***Porphyrin and chlorophyll metabolism*** | | | | | | | | |
| 1 | 101218189 | Divinyl chlorophyllide a 8-vinyl-reductase | 1.3.1.75 | 1.44 | 0.69 |  | 0.77 | 1.30 |
| 2 | 101203653 | Chlorophyllase-2 | 3.1.1.14 | 1.80 | 0.56 |  | 0.98 | 1.02 |
| 3 | 101206296 | Protein STAY-GREEN homolog | 4.99.1.10 | 1.46 | 0.68 |  | 0.72 | 1.38 |
| 4 | 101206600 | Magnesium protoporphyrin IX methyltransferase | 2.1.1.11 | 1.54 | 0.65 |  | 0.61 | 1.63 |
| 5 | 101207399 | Glutamate-1-semialdehyde 2,1-aminomutase 2 | 5.4.3.8 | 1.45 | 0.69 |  | 0.84 | 1.19 |
| 6 | 101208123 | 7-Hydroxymethyl chlorophyll a reductase | 1.17.7.2 | 1.90 | 0.53 |  | 0.91 | 1.10 |
| 7 | 101208135 | Glutamyl-tRNA reductase 2 | 1.2.1.70 | 0.97 | 1.03 |  | 1.24 | 0.80 |
| 8 | 101208461 | Magnesium-chelatase subunit (*ChlD*) | 6.6.1.1 | 1.47 | 0.68 |  | 0.64 | 1.56 |
| 9 | 101209548 | Oxygen-dependent coproporphyrinogen-III oxidase | 1.3.3.3 | 0.94 | 1.06 |  | 0.78 | 1.28 |
| 10 | 101212175 | Chlorophyll synthase | 2.5.1.62 2.5.1.133 | 0.90 | 1.11 |  | 0.81 | 1.23 |
| 11 | 101212208 | Uroporphyrinogen decarboxylase 1 | 4.1.1.37 | 1.08 | 0.92 |  | 0.83 | 1.20 |
| 12 | 101212226 | Chlorophyll(ide) b reductase (*NOL*) | 1.1.1.294 | 0.59 | 1.71 |  | 0.92 | 1.08 |
| 13 | 101213181 | Protoporphyrinogen oxidase | 1.3.3.4 1.3.3.15 | 1.00 | 1.00 |  | 0.83 | 1.20 |
| 14 | 101213619 | Magnesium-chelatase subunit (*ChlH*) | 6.6.1.1 | 2.39 | 0.42 |  | 1.16 | 0.86 |
| 15 | 101214191 | Heme oxygenase 1 | 1.14.15.20 | 1.66 | 0.60 |  | 0.79 | 1.27 |
| 16 | 101214667 | Protoporphyrinogen oxidase 1 | 1.3.3.4 1.3.3.15 | 1.29 | 0.77 |  | 1.16 | 0.86 |
| 17 | 101217637 | Magnesium-chelatase subunit (*ChlI*) | 6.6.1.1 | 1.14 | 0.87 |  | 0.90 | 1.11 |
| 18 | 101217650 | Phytochromobilin:ferredoxin oxidoreductase | 1.3.7.4 | 1.35 | 0.74 |  | 1.02 | 0.98 |
| 19 | 101218513 | Glutamate--tRNA ligase | 6.1.1.17 | 0.96 | 1.04 |  | 0.89 | 1.12 |
| 20 | 101219179 | Magnesium-protoporphyrin IX monomethyl ester [oxidative] cyclase | 1.14.13.81 | 2.37 | 0.42 |  | 0.73 | 1.36 |
| 21 | 101219336 | Magnesium dechelatase (*SGRL*) | 4.99.1.10 | 0.72 | 1.38 |  | 0.58 | 1.72 |
| 22 | 101219749 | Porphobilinogen deaminase | 2.5.1.61 | 0.90 | 1.12 |  | 0.69 | 1.45 |
| 23 | 101220615 | Glutamyl-tRNA reductase 1 | 1.2.1.70 | 1.36 | 0.73 |  | 0.41 | 2.42 |
| 24 | 101221755 | Geranylgeranyl diphosphate reductase | 1.3.1.83 | 1.65 | 0.61 |  | 0.63 | 1.58 |
| 25 | 101222061 | Uroporphyrin-III *C*-methyltransferase | 2.1.1.107 | 0.32 | 3.10 |  | 1.32 | 0.76 |
| 26 | 101222312 | *Delta*-aminolevulinic acid dehydratase | 4.2.1.24 | 1.19 | 0.84 |  | 0.74 | 1.35 |
| 27 | 101222607 | Protoheme IX farnesyltransferase | 2.5.1.141 | 0.74 | 1.35 |  | 0.98 | 1.02 |
| 28 | 101222986 | Probable chlorophyll(ide) b reductase (*NYC1*) | 1.1.1.294 | 0.98 | 1.02 |  | 1.19 | 0.84 |
| 29 | 101223042 | Sirohydrochlorin ferrochelatase | 4.99.1.4 | 1.56 | 0.64 |  | 0.89 | 1.12 |
| 30 | 101223133 | Red chlorophyll catabolite reductase | 1.3.7.12 | 1.87 | 0.53 |  | 0.77 | 1.30 |
| 31 | 101208554 | NPR; protochlorophyllide reductase | 1.3.1.33 | 1.55 | 0.65 |  | 0.44 | 2.29 |
| ***Carotenoid biosynthesis*** | | | | | | | | |
| 32 | 101212508 | Abscisic acid 8'-hydroxylase (CYP707A2) | 1.14.14.137 | 0.41 | 2.46 |  | 2.92 | 0.34 |
| 33 | 101204647 | 9-cis-Epoxycarotenoid dioxygenase (*NCED2*) | 1.13.11.51 | 0.94 | 1.06 |  | 1.41 | 0.71 |
| 34 | 101204961 | Carotene epsilon-monooxygenase | 1.14.14.158 | 1.22 | 0.82 |  | 1.04 | 0.96 |
| 35 | 101206471 | Phytoene synthase | 2.5.1.32 | 0.95 | 1.05 |  | 1.44 | 0.69 |
| 36 | 101207778 | Lycopene *beta*-cyclase | 5.5.1.19 | 1.86 | 0.54 |  | 1.14 | 0.88 |
| 37 | 101210522 | ζ-carotene desaturase | 1.3.5.6 | 1.15 | 0.87 |  | 0.68 | 1.47 |
| 38 | 101211624 | 15-*cis*-ζ-carotene isomerase | 5.2.1.12 | 1.36 | 0.73 |  | 0.68 | 1.47 |
| 39 | 101212140 | β-carotene hydroxylase 2 | 1.14.15.24 | 0.76 | 1.31 |  | 1.50 | 0.67 |
| 40 | 101212850 | Prolycopene isomerase | 5.2.1.13 | 1.38 | 0.72 |  | 0.95 | 1.05 |
| 41 | 101213753 | Lycopene epsilon cyclase | 5.5.1.18 | 0.91 | 1.10 |  | 0.61 | 1.63 |
| 42 | 101214325 | β-carotene 3-hydroxylase 1 | 1.14.15.24 | 0.61 | 1.63 |  | 1.76 | 0.57 |
| 43 | 101214823 | Phytoene synthase 2 | 2.5.1.32 | 2.35 | 0.43 |  | 0.88 | 1.13 |
| 44 | 101215492 | Protein LUTEIN DEFICIENT 5 | 1.14.-.- | 0.71 | 1.42 |  | 0.82 | 1.22 |
| 45 | 101218773 | Carotenoid cleavage dioxygenase 7 | 1.13.11.68 | 0.43 | 2.31 |  | 0.89 | 1.12 |
| 46 | 101219340 | β-carotene isomerase D27 | 5.2.1.14 | 0.55 | 1.83 |  | 0.98 | 1.02 |
| 47 | 101219413 | Xanthoxin dehydrogenase | 1.1.1.288 | 1.90 | 0.53 |  | 0.89 | 1.12 |
| 48 | 101220226 | Probable carotenoid cleavage dioxygenase 4 | 1.13.11.51 | 1.00 | 1.00 |  | 0.98 | 1.02 |
| 49 | 101214535 | 9-*cis*-Epoxycarotenoid dioxygenase (*NCED2*) | 1.13.11.51 | 1.18 | 0.85 |  | 0.65 | 1.53 |
| 50 | 101219505 | 9-*cis*-Epoxycarotenoid dioxygenase (*NCED3*) | 1.13.11.51 | 3.54 | 0.28 |  | 1.27 | 0.79 |
| 51 | 101216870 | Violaxanthin de-epoxidase | 1.23.5.1 | 1.32 | 0.76 |  | 0.84 | 1.18 |
| 52 | 101212903 | Zeaxanthin epoxidase | 1.14.15.21 | 1.97 | 0.51 |  | 1.01 | 0.99 |
| ***Flavonoid biosynthesis*** | | | | | | | | |
| 53 | 101204747 | Cytochrome P450 (CYP73A100) | 1.14.14.91 | 1.26 | 0.79 |  | 2.95 | 0.34 |
| 54 | 101205211 | Chalcone synthase 2 | 2.3.1.74 | 1.83 | 0.55 |  | 0.74 | 1.36 |
| 55 | 101205537 | Cytochrome P450 (CYP73A100) | 1.14.14.91 | 2.13 | 0.47 |  | 3.60 | 0.28 |
| 56 | 101205648 | Caffeoyl-CoA *O*-methyltransferase 5 | 2.1.1.104 | 2.66 | 0.38 |  | 1.59 | 0.63 |
| 57 | 101206443 | Naringenin, 2-oxoglutarate 3-dioxygenase | 1.14.11.9 | 1.29 | 0.78 |  | 1.38 | 0.72 |
| 58 | 101206618 | Stemmadenine *O*-acetyltransferase | 2.3.1.133 | 0.93 | 1.08 |  | 1.00 | 1.00 |
| 59 | 101212121 | Putative caffeoyl-CoA *O*-methyltransferase (At1g67980) | 2.1.1.104 | 0.65 | 1.54 |  | 0.86 | 1.16 |
| 60 | 101214857 | Shikimate *O*-hydroxycinnamoyltransferase | 2.3.1.133 | 0.47 | 2.11 |  | 0.70 | 1.42 |
| 61 | 101217257 | Cytochrome P450 (98A2) | 1.14.14.96 | 1.46 | 0.68 |  | 3.73 | 0.27 |
| 62 | 101219245 | Flavonol synthase/flavanone 3-hydroxylase | 1.14.20.6 | 1.56 | 0.64 |  | 1.20 | 0.83 |
| 63 | 101221846 | Trans-cinnamate 4-monooxygenase | 1.14.14.91 | 0.96 | 1.04 |  | 1.09 | 0.92 |
| 64 | 105434566 | Cytochrome P450 (CYP73A100) | 1.14.14.91 | 1.47 | 0.68 |  | 3.50 | 0.29 |
| 65 | 101203846 | phenylalanine ammonia-lyase | 4.3.1.24 | 1.84 | 0.54 |  | 2.59 | 0.39 |
| 66 | 101204417 | 4-coumarate-CoA ligase | 6.2.1.12 | 1.09 | 0.92 |  | 0.73 | 1.37 |

Genes were selected by *p* < 0.05.; ^a^Biological information in sequences.; ^b^Enzyme commission number for enzyme from KEGG database (http://www.genome.jp/kegg/).; The colored squares (blue-to-red) represent fold changes normalized by the average of each gene expression of Chuichung and Mini. The color scheme is as follows: Lower limit value, 0.7 (blue); middle limit value, 1 (white); upper limit value, 1.3 (red).

**Reference**

Abu-Reidah, I. M., Arráez-Román, D., Quirantes-Piné, R., Fernández-Arroyo, S., Segura-Carretero, A., and Fernández-Gutiérrez, A. (2012). HPLC-ESI-Q-TOF-MS for a comprehensive characterization of bioactive phenolic compounds in cucumber whole fruit extract. *Food Res. Int.* 46, 108–117. doi:10.1016/j.foodres.2011.11.026.

Abu Reidah, I. M. (2013). Characterization of Phenolic Compounds in Highly-Consumed Vegetable Matrices By Using Advanced Analytical Techniques.

Brito, A., Ramirez, J. E., Areche, C., Sepúlveda, B., and Simirgiotis, M. J. (2014). HPLC-UV-MS profiles of phenolic compounds and antioxidant activity of fruits from three *citrus* species consumed in Northern Chile. *Molecules* 19, 17400–17421. doi:10.3390/molecules191117400.

Chen, G., Song, C., Jin, S., Li, S., Zhang, Y., Huang, R., et al. (2017). An integrated strategy for establishment of metabolite profile of endogenous lysoglycerophospholipids by two LC-MS/MS platforms. *Talanta* 162, 530–539. doi:10.1016/j.talanta.2016.10.045.

Du, L. Y., Zhao, M., Xu, J., Qian, D. W., Jiang, S., Shang, E. X., et al. (2014). Analysis of the metabolites of isorhamnetin 3-*O*-glucoside produced by human intestinal flora in vitro by applying ultraperformance liquid chromatography/quadrupole time-of-flight mass spectrometry. *J. Agric. Food Chem.* 62, 2489–2495. doi:10.1021/jf405261a.

Kitajima, J., Kamoshita, A., Ishikawa, T., Takano, A., Fukuda, T., Isoda, S., et al. (2003). Glycosides of *Atractylodes lancea*. *Chem. Pharm. Bull.* 51, 673–678. doi:10.1248/cpb.51.673.

Lévèques, A., Actis-Goretta, L., Rein, M. J., Williamson, G., Dionisi, F., and Giuffrida, F. (2012). UPLC-MS/MS quantification of total hesperetin and hesperetin enantiomers in biological matrices. *J. Pharm. Biomed. Anal.* 57, 1–6. doi:10.1016/j.jpba.2011.08.031.

Moco, S., Bino, R. J., Vorst, O., Verhoeven, H. A., De Groot, J., Van Beek, T. A., et al. (2006). A liquid chromatography-mass spectrometry-based metabolome database for tomato. *Plant Physiol.* 141, 1205–1218. doi:10.1104/pp.106.078428.

Santos, J., Mendiola, J. A., Oliveira, M. B. P. P., Ibáñez, E., and Herrero, M. (2012). Sequential determination of fat- and water-soluble vitamins in green leafy vegetables during storage. *J. Chromatogr. A* 1261, 179–188. doi:10.1016/j.chroma.2012.04.067.

Serafini, M., Bugianesi, R., Salucci, M., Azzini, E., Raguzzini, A., and Maiani, G. (2002). Effect of acute ingestion of fresh and stored lettuce ( *Lactuca sativa* ) on plasma total antioxidant capacity and antioxidant levels in human subjects. *Br. J. Nutr.* 88, 615–623. doi:10.1079/bjn2002722.

Suárez-García, S., Arola, L., Pascual-Serrano, A., Arola-Arnal, A., Aragonès, G., Bladé, C., et al. (2017). Development and validation of a UHPLC-ESI-MS/MS method for the simultaneous quantification of mammal lysophosphatidylcholines and lysophosphatidylethanolamines in serum. *J. Chromatogr. B Anal. Technol. Biomed. Life Sci.* 1055–1056, 86–97. doi:10.1016/j.jchromb.2017.04.028.

Ul Haq, F., Ali, A., Khan, M. N., Shah, S. M. Z., Kandel, R. C., Aziz, N., et al. (2019). Metabolite Profiling and Quantitation of Cucurbitacins in Cucurbitaceae Plants by Liquid Chromatography coupled to Tandem Mass Spectrometry. *Sci. Rep.* 9, 1–11. doi:10.1038/s41598-019-52404-1.

Zhao, L., Huang, Y., Zhou, H., Adeleye, A. S., Wang, H., Ortiz, C., et al. (2016). GC-TOF-MS based metabolomics and ICP-MS based metallomics of cucumber (: *Cucumis sativus*) fruits reveal alteration of metabolites profile and biological pathway disruption induced by nano copper. *Environ. Sci. Nano* 3, 1114–1123. doi:10.1039/c6en00093b.
